# Non-Penicillin-Susceptible *Streptococcus suis* Isolated from Humans

**DOI:** 10.3390/pathogens10091178

**Published:** 2021-09-13

**Authors:** Nichari Bamphensin, Peechanika Chopjitt, Rujirat Hatrongjit, Parichart Boueroy, Nahuel Fittipaldi, Marcelo Gottschalk, Anusak Kerdsin

**Affiliations:** 1 Faculty of Public Health, Kasetsart University Chalermphrakiat Sakon Nakhon Province Campus, Sakon Nakhon 47000, Thailand; nicharee905@gmail.com (N.B.); peechanika.c@ku.th (P.C.); parichart.bou@ku.th (P.B.); 2 Department of General Sciences, Faculty of Science and Engineering, Kasetsart University Chalermphrakiat Sakon Nakhon Province Campus, Sakon Nakhon 47000, Thailand; rujirat.ha@ku.th; 3 GREMIP, Faculty of Veterinary Medicine, University of Montreal, Montreal, QC H3T 1J4, Canada; n.fittipaldi@umontreal.ca (N.F.); marcelo.gottschalk@umontreal.ca (M.G.)

**Keywords:** *Streptococcus suis*, antimicrobial, penicillin, sequence type, serotype, genome

## Abstract

*Streptococcus suis* is a pathogen that causes invasive infections in humans and pigs. In this study, 448 *S. suis* isolates recovered from human infections in Thailand were characterized with regard to their antimicrobial susceptibility and antimicrobial resistance genes, including, for non-penicillin-susceptible isolates, sequence analyses of five genes encoding penicillin-binding proteins (*pbp1a, pbp1b, pbp2a, pbp2b*, and *pbp2x*). All 448 isolates were susceptible to cefepime and ceftriaxone, whereas 99.6%, 91.7%, and 72.9% of the isolates were susceptible to levofloxacin, penicillin, and chloramphenicol, respectively. Almost all isolates were resistant to tetracycline (98.2%), clindamycin (94%), erythromycin (92.4%), and azithromycin (82.6%). Genes *tet*(O) and *ermB* were the predominant resistance genes detected among macrolide- and tetracycline-resistant isolates. A total of 37 out of 448 isolates (8.2%) showed intermediately resistance to penicillin. Most of these isolates (59.5%) belonged to serotype 2-ST233. Comparison of the predicted translated sequences of five PBP proteins of a penicillin-susceptible isolate (strain P1/7) to the respective PBP sequences of ten non-penicillin-susceptible isolates revealed multiple amino acid substitutions. Isolates of CC221/234 showed highly variable amino acid substitutions in all PBP proteins. An ST104 isolate had a higher number of amino acid substitutions in PBP2X. Isolates belonging to CC233/379 had numerous substitutions in PBP2B and PBP2X. ST25 isolates exhibited fewer amino acid substitutions than isolates of other STs in all five PBPs. The antimicrobial resistance of *S. suis* is increasing worldwide; therefore, restrictions on antimicrobial use, continuous control, and the surveillance of this bacterium throughout the pork supply chain are crucial for ensuring public health and must be a priority concern.

## 1. Introduction

*Streptococcus suis*, an important zoonotic pathogen, causes invasive infections in humans in close contact with infected pigs or contaminated pork-derived products [1]. The number of reported human cases, especially in Southeast Asian countries, has dramatically increased in the past few years [1,2]. Among the 29 serotypes of *S. suis*, serotype 2 is the most common cause of human infections, although human cases due to serotypes 4, 5, 9, 14, 16, 21, 24, and 31 have also been reported [1,3,4,5,6,7,8,9].

Although some have raised the alarm about the risks posed by increasing antimicrobial resistance in *S. suis*, including acquisition/transmission and the organism serving as a reservoir for resistance genes, *S. suis* has not yet been broadly acknowledged as a concern by the medical community [10,11]. One reason for this is that antimicrobial resistance data for *S. suis* continue to be scarce. Furthermore, most available antimicrobial resistance data have been generated by investigating *S. suis* isolates recovered from pigs [11,12,13,14,15,16,17], whereas those for human *S. suis* isolates are minimal [16,18,19,20]. The mass use of antibiotics in the livestock industry in many countries, especially in pigs for growth promotion, routine prophylaxis, or treatment for controlling the spread of infection, has potentially led to antimicrobial resistance in the microbial communities of food animal production under such conditions [2].

Here, we report the antimicrobial susceptibility and resistance gene profiles of 448 *S. suis* isolates of different serotypes and genetic backgrounds recovered from human infections in Thailand. We also sequenced the genomes of non-penicillin-susceptible isolates to begin to reveal the molecular reasons for decreased penicillin susceptibility in *S. suis*.

## 2. Results 

### 2.1. Antimicrobial Susceptibility 

**Figure 1:**
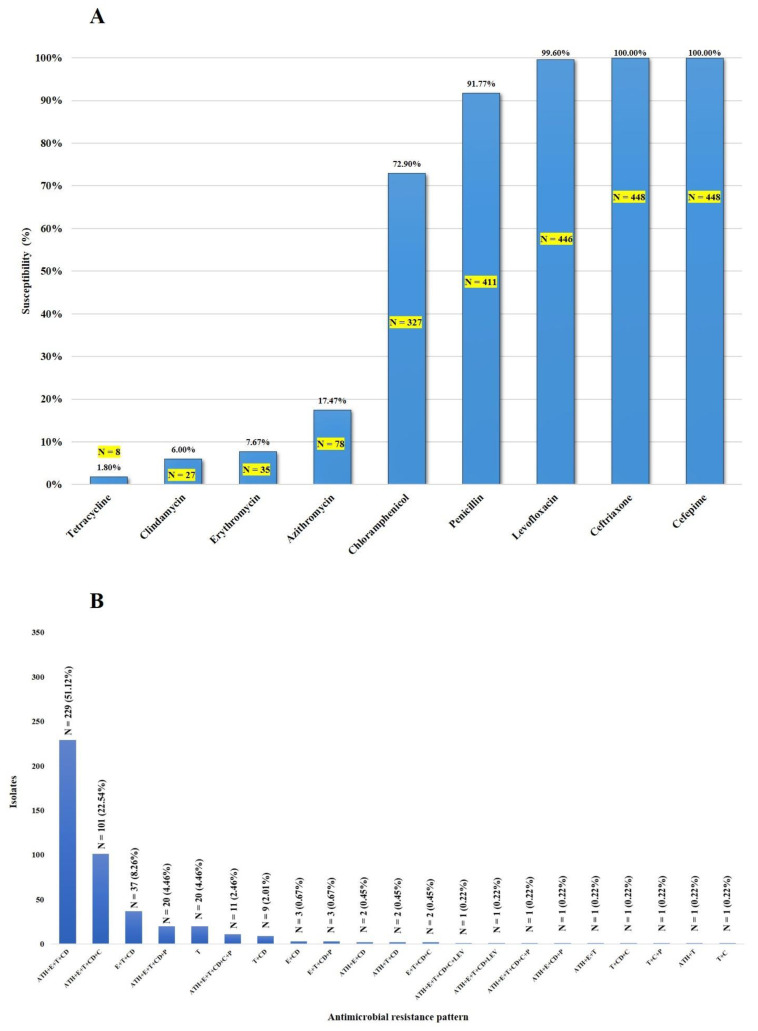
Susceptibility of *S. suis* isolates to antimicrobial agents (**A**) and the distribution of antimicrobial resistance profiles of *S. suis* isolates (**B**).

As shown in Figure 1A, all 448 isolates (one isolate/patient) were susceptible to cefepime and ceftriaxone, whereas 99.6%, 91.7%, and 72.9% were susceptible to levofloxacin, penicillin, and chloramphenicol, respectively. Thirty-seven isolates (8.3%) showed intermediate resistance to penicillin (Table 1). Among these non-penicillin-susceptible isolates, serotype 2-ST233 was predominant (22/37; 59.5%). The vast majority of the isolates were resistant to tetracycline (98.2%), clindamycin (94%), erythromycin (92.4%), and azithromycin (82.6%). As shown in Figure 1B and Table S1, the macrolide-lincosamide–tetracycline (azithromycin (ATH)-erythromycin (E)-clindamycin (CD)-tetracycline-T) resistance pattern was the most frequently found (51.1%), followed by macrolide-lincosamide-tetracycline-phenicol (azithromycin (ATH)-erythromycin (E)-clindamycin (CD)-tetracycline (T)-chloramphenicol (C)) (22.5%). Of the 448 isolates, 410 (91.5%) were considered to be multidrug-resistant (MDR), as shown in Figure 1B. This study demonstrates that some *S. suis* isolates exhibit non-susceptibility to five antimicrobial categories (at least one agent in each category), either macrolide-lincosamide-tetracycline-chloramphenicol-penicillin (*N* = 10) or macrolide-lincosamide-tetracycline-chloramphenicol-levofloxacin (*N* = 1), as shown in Figure 1B. All 37 non-penicillin-susceptible isolates exhibited the MDR phenotype (Table S1).

Information about the antibiotic treatments of patients was available for 445 isolates (Table S2). Indeed, 95.7% of cases were treated with ceftriaxone and 4.3% were treated with a combination of penicillin and ceftriaxone. According to antimicrobial susceptibility results, all of these isolates were susceptible to ceftriaxone and cefepime that belonged to third-generation cephalosporins.

### 2.2. Macrolide- and Tetracycline-Resistance Genes

Table 2 shows that *ermB* was the most commonly detected macrolide-resistance gene among the isolates (*N* = 384; 85.7%). None of the isolates carried *mef (A/E)* and *msrD*. However, 64 isolates (14.3%) were phenotypically resistant to macrolides, although *ermB, mef* *(A/E)*, or *msrD* were not detected, suggesting that the macrolide resistance exhibited by these isolates is due to other mechanisms. In the case of tetracycline-resistance genes, gene *tet* (O) was the most commonly identified (*N* = 409, 91.3%). A total of 26 isolates (5.8%) carried *tet* (O) + *tet* (W). Other *tet* genes detected were *tet* (M), *tet* (M)+*tet* (L), and *tet* (W). Nine isolates (2%) had a tetracycline-resistant phenotype, but we did not detect *tet, tcr3*, *otrB*, or *otrC* genes by PCR in these organisms. Among the macrolide- and tetracycline-resistance genes detected in 410 MDR *S. suis* isolates, *ermB*+*tet* (O) was predominant, found in 337 (82.2%) isolates (Table S1). Only *tet* (O) was found in 39 (9.5%) isolates, and *ermB* + *tet* (O) + *tet* (W) was detected in 24 (5.9%) isolates. The remaining MDR *S. suis* isolates exhibited *ermB* for 6 (1.5%) isolates, *tet* (L) + *tet* (M) for 2 (0.5%) isolates, and one isolate each for *tet* (O) + *tet* (W) and only *tet* (W), as shown in Table S1.

### 2.3. Whole-Genome Analysis of Non-penicillin-Susceptible S. suis Isolates

Of the 37 non-penicillin-susceptible isolates, 10 were selected for whole-genome sequence analysis, including 4 ST233 isolates and 1 isolate each of ST25, ST104, ST105, ST221, ST234, and ST379. Resfinder and CARD were used to identify antimicrobial resistance genes in these isolates (Table 3). Genes *ermB* and *tet* (O) were found in all 10 isolates. Additional macrolide resistance genes included gene *Inu (A)*, detected in ST105 (serotype 14; ID32494), whereas genes *Inu (B)* and *Isa (E)* were found in ST104 (serotype 2; ID27715). In addition, this ST104 also carried aminoglycoside-resistance genes *aac (6′)-aph (2″)* and *aph (3′)-III* (Table 3). 

Analysis of plasmid replicon types with PlasmidFinder identified a rep21-type replicon only in the ST105 isolate. However, PLACNETw analysis revealed that MOBP and MOPT replicon types were frequently detected in our isolates (*N* = 8 in both cases). Interestingly, isolates in CC104 and CC233/379 had the same MOB replicon types, whereas ST221 and ST234 exhibited MOBP and MOBV. ST25 carried plasmid replicon types MOBV and MOBT. ST235 (serotype 5) had three MOB replicon types (MOBP, MOBV, and MOBT). It was apparent that MOB type plasmids were specific to ST or CC. A summary of the plasmid replicon types is shown in Table 3.

Analysis of five *pbp* genes (*pbp1a, pbp1b, pbp2a, pbp2b*, and *pbp2x*) of ten penicillin-non-susceptible isolates compared to *S. suis* P1/7 (penicillin-susceptible isolate) revealed multiple nucleotide substitutions impacting primary protein sequences throughout all *pbp* genes (Table 3 and Supplementary Files 1–5). In contrast with other isolates, strains ID33329 (ST221) and ID32098 (ST234) of CC221/234 had highly variable numbers of amino acid substitutions. Isolate ID27715 (ST104) had higher rates of amino acid substitutions in the predicted translated sequence of *pbp2x* than in other *pbp* gene-encoded proteins. Isolates in CC233/379 had high amino acid substitutions in the predicted *pbp2b* and *pbp2x* gene products. Isolate ID30190 (ST25) exhibited few amino acid substitutions in the translated sequences of all five *pbp* genes. In contrast, we did not identify substitutions in an ST105 isolate (ID32494), although it displayed intermediate resistance.

**Figure 2:**
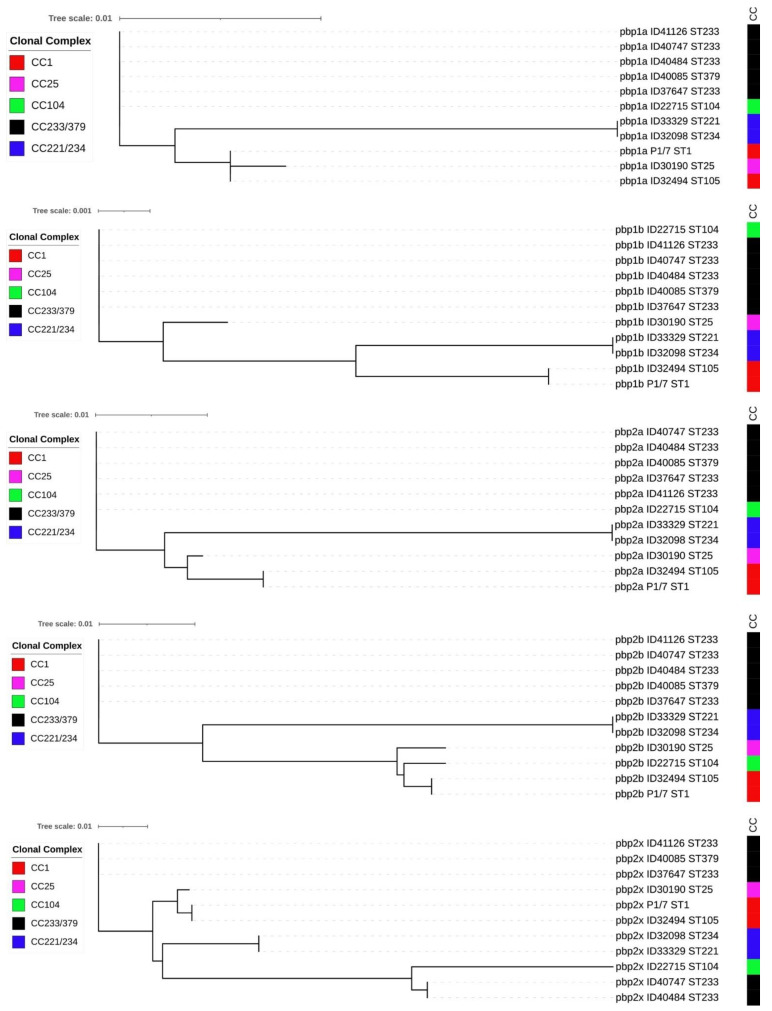
Phylogenetic tree of 5 PBP amino acid sequences of 10 non-penicillin-susceptible *S. suis* isolates and penicillin-susceptible *S. suis* P1/7.

In addition, insertions of amino acids were detected between amino acid positions 708 and 709 in PBP1A of isolate ID32098 (ST234) and ID33329 (ST221), and between positions 433 and 434 of PBP2B for isolate ID30190 (ST25) (Supplementary Files 1 and 4). We also detected deletions of amino acid in the PBP2B of seven isolates at position 433 and two isolates at position 672 (Supplementary File 4). Phylogenetic trees were constructed using the PBP amino acid sequences of these non-penicillin-susceptible isolates (Figure 2). Five of these phylogenetic trees showed different clustering of the isolates, which were concordant with STs. ST104 (CC104), ST233 (CC233/379), and ST379 (CC233/379) isolates clustered together in the phylogenetic trees of PBP1A, PBP1B, PBP2A, and PBP2B, although they belong to different clonal complex. However, the PBP2X tree revealed that some isolates of ST233 and ST379 clustered separately from ST104 and ST233 isolates (Figure 2).

**Figure 3:**
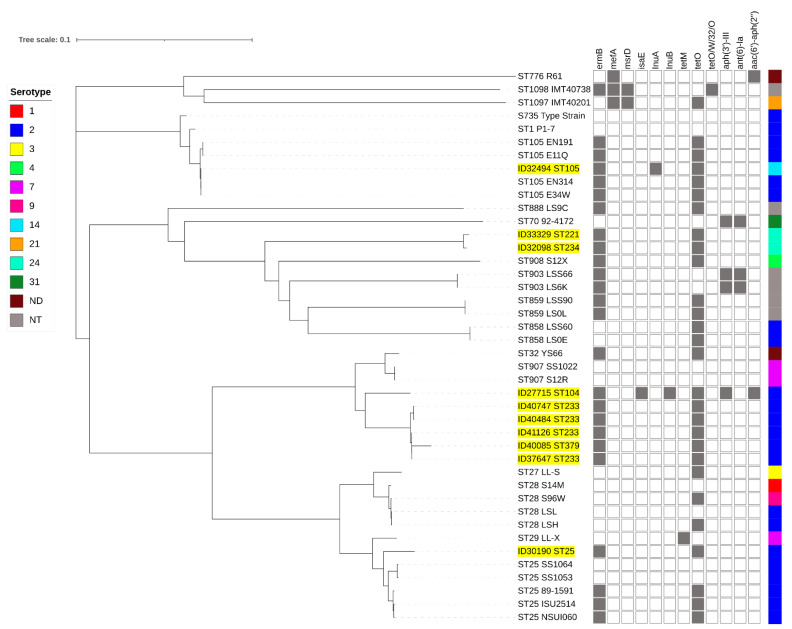
Whole-genome phylogeny analysis of *S. suis* generated by CSI phylogeny and visualized with an interactive life tool tree. *S. suis* strains used in this study are highlighted in yellow. Presentation of the antimicrobial-resistance genes in each *S. suis* strain is shown by filled squares. ND, serotype is not determined; NT, non-typable.

Full-genome SNP-based phylogenetic analysis showed that the penicillin-non-susceptible isolates were distributed throughout the phylogenetic tree (Figure 3). However, they were clustered accordingly to their STs or CCs. Isolates of STs 104 (ID27715), 233 (ID37647, ID40484, ID40747, and ID41126), and 379 (ID40085) clustered together, whereas ID30190-ST25 clustered with other ST25 isolates. ID32494 (ST105) clustered with CC1 isolates. The serotype 14-ST105 (ID32494) isolate was very closely related to serotype 2-ST105 isolates (E34W, EN314, E11Q, and EN191) originating from Vietnam. ST221 (ID33329) and ST234 (ID32098) isolates were very closely related to ST903, 908, 858, and 859 isolates.

## 3. Discussion

*S. suis* infections have a significant economic impact on the swine industry and are also a public health concern, with a multitude of reported human infections in Southeast Asian countries [1,11]. Other than the loss of lives, and frequent sequalae, a study in Thailand recently showed that *S. suis* human infections are responsible for an estimated USD 11.3 million loss in productivity-adjusted life years in the gross domestic product, which equates to USD 36,033 lost per person [21]. In addition to the economic burden, this bacterium is one of the main reasons for antimicrobial use in swine farms for routine prophylaxis or metaphylaxis, and a reservoir of antimicrobial-resistant genes [2,11]. Antimicrobial resistance is a major health problem; data suggest an increasing and alarming contribution of *S. suis* to this global threat [2].

Worldwide antimicrobial resistance data available for *S. suis* indicate that most isolates recovered from both humans and pigs have high resistance to tetracycline and moderate to high resistance to macrolides, e.g., erythromycin [12,13,14,15,16,17,19,22]. Our study confirms those observations. In addition, our data show that clindamycin (lincosamide) resistance was very common (94%) among *S. suis* human isolates characterized here. Other studies have shown a wide range of rates of resistance to clindamycin in *S. suis* isolates from pigs (38.5–98.7%) and humans (23.8–81.5%) [12,14,15,16,17,18,23]. On the other hand, a study from The Netherlands showed very low levels of *S. suis* resistance to clindamycin (0.5%) [24]. This may be due to differences in the usage of antimicrobials between Asia and Europe: there is highly extensive use in Asian countries but low levels of use in European countries [25,26].

Tetracycline-resistance gene presence in *S. suis* is variable. Genes *tet* (B), *tet* (L), *tet* (M), *tet* (O), *tet* (S), *tet* (W), *tet* (40), *tet* (O/32/O), and *tet* (O/W/32/O) have been reported [12,14,17,19,27,28]. Of these *tet* genes, *tet*(O), encoding the ribosome protection protein, is the most frequently described gene in *S. suis* isolates from pigs and humans worldwide [12,14,17,18,22,27,28]. In agreement with these previous findings, the *tet* (O) gene was the predominant *tet* gene among our isolates. Resistance to macrolides is mainly due to the action of erythromycin ribosomal methylases encoded by *erm* genes or to macrolide efflux pumps encoded by *mef* genes [22]. In this study, *ermB* (85.7%) was the most frequently detected macrolide gene, replicating previous findings in *S. suis* isolates recovered from both pigs and humans [14,17,18,22,23,27,28]. This gene could contribute to lincosamide and streptogamin B resistance due to the detected clindamycin resistance in *S. suis* isolates. However, 14.3% of *S. suis* in our study did not harbor *ermB, mef (A/E),* or *msrD* genes, suggesting that, similarly to what has been reported in a previous study [12], other mechanisms such as 23S rRNA methyl transferase (*cfr*) or macrolide phosphotransferase (*mph*(*C*)) are responsible for the macrolide-resistant phenotype displayed by the isolates analyzed here [22].

Despite the longstanding worldwide usage of *β*-lactams in pigs, the majority of *S. suis* isolates remain susceptible to this class of antibiotics. In this investigation, all human *S. suis* isolates were susceptible to ceftriaxone and cefepime, and most were susceptible to penicillin. However, we detected penicillin non-susceptibility (intermediate resistance) in a relatively significant percentage (8.3%) of our clinical isolates. Although several reports have shown different resistance rates to penicillin (from 0.5 up to as high as 62%) among *S. suis* isolates from pigs [15,16,17,23,24,29], our data differ greatly from what has been described thus far in terms of penicillin non-susceptibility for other human *S. suis* isolates [16,18,19,20]; therefore, our results are alarming. 

Our data show that isolates belonging to serotype 2 ST233 were the most common strain type associated with penicillin non-susceptibility. Serotype 14 (ST105) and serotype 24 (ST221 and ST234) were also found to be intermediately resistant to penicillin. Previous studies have reported penicillin non-susceptibility in serotypes 2, 7, and 21 (ST1097), as well as in non-serotypeable (ST776, ST1098) isolates [15,16,17,23,30]. The mechanisms of *β*-lactam resistance in *S. suis* are not fully known. Our data expand on previous findings that analyzed four *pbp* genes (*pbp2x, pbp2b, pbp1a,* and *pbp2a*) in *S. suis* strain R61, and discovered the presence of multiple amino acid substitutions throughout the entire sequence of the predicted translated protein sequences of these genes [31]. Substitutions were mostly high in the PBP2X and PBP2B proteins, which are the targets for resistance to *β*-lactam antibiotics, similar to previous reports [30,31]. Mutations in PBP proteins may affect enzyme catalysis, binding-site affinity, stability, or structural configuring changes that lead to resistance to the antibiotic. However, our *S. suis* serotype 14 (ST105), which was intermediately resistant to penicillin, did not present any substitutions all five PBP proteins, suggesting that other mechanisms are involved. Indeed, as shown in *S*. *pneumoniae* and *S. gordonii*, penicillin non-susceptibility may occur via the involvement of other factors, such as a putative glycosyltransferase (*cpoA*), a histidine protein kinase (*ciaH*), and/or a putative iron permease (spr1178) [32,33,34].

Our phylogenetic analysis of five PBP proteins showed that isolate clusters were concordant with STs or CCs. This seems to indicate that the diversity of the PBP proteins is associated with STs or CCs. Therefore, the diversity of five PBP proteins should be extensively compared and analyzed using isolates belonging to different STs or serotypes in the future. Similarly, an SNP-based phylogenetic tree based on whole-genome analysis of our 10 non-penicillin-susceptible isolates, and closely related strains, showed that isolate clustering followed grouping by STs or CCs. Based on the MLST database, the cluster of STs104/233/379 was closely related to ST907 (serotype 7) which was isolated from pigs in the United Kingdom. ST221/234 was related to ST908 (serotype 4 and 9), ST903 (non-serotypeable), ST858 (serotype 2), and ST859 (non-serotypeable), which all were isolated from pigs in the United Kingdom. It is interesting that our serotype 14-ST105 isolate was closely related to serotype 2-ST105 isolates from Vietnam, which may suggest capsular gene switching [35].

## 4. Materials and Methods 

### 4.1. Bacterial Isolates

A total of 448 isolates of *S. suis* from human specimens and belonging to different collection dates, serotypes, sequence types (STs), and regions in Thailand were included in the current study (Table S2). Serotypes and STs of these selected isolates were already known [5,6,7,8,36,37]. Of 448 isolates, 392 were serotype 2 and 49 were serotype 14, whereas the rest were serotype 24 (*N* =3), serotype 5 (*N* = 2), serotype 4 (*N* = 1), and serotype 9 (*N* = 1). Details of these isolates and their origins are shown in Tables S1 and S2.

### 4.2. Antimicrobial Susceptibility Testing

The broth microdilution technique was used according to the standards defined in the Clinical and Laboratory Standard Institute (CLSI) guidelines 2020 (M100-30th edn; CLSI, 2020) to determine the minimum inhibitory concentrations (MICs) of penicillin (≤0.12 μg/mL = Susceptible; 0.25–2 μg/mL = Intermediate; ≥4 μg/mL = Resistance) and ceftriaxone (≤1 μg/mL = Susceptible; 2 μg/mL = Intermediate; ≥4 μg/mL = Resistance) [38]. Susceptibility to other antimicrobials, such as cefepime, azithromycin, erythromycin, tetracycline, clindamycin, levofloxacin, and chloramphenicol, were determined using a disk diffusion technique following the 2020 CLSI-M100 guidelines [38]. There are currently no breakpoints recommended for *S. suis*; therefore, those for viridans group streptococci were used, as defined in the 2020 CLSI-M100 guidelines [38]. *Streptococcus pneumoniae* strain ATCC 49619 was used for quality control purposes.

### 4.3. PCR Detection of Macrolide- and Tetracycline-Resistance Genes

Previously described PCR assays were used to detect three macrolide-resistance genes (*ermB, mefA,* and *msrD*) and 43 tetracycline-resistance genes (Table S3) [27,39].

### 4.4. Whole-Genome Sequencing (WGS)

The genomes of ten non-penicillin-susceptible *S. suis* isolates were further selected for WGS in order to investigate nucleotide variation in *pbp* genes. These isolates were chosen based on their different serotypes (2, 14, and 24) and STs, including four ST233 isolates, and one representative isolate each of ST25, ST104, ST105, ST221, ST234, and ST379. WGS was carried out on the Illumina platform at MicrobesNG (Birmingham, UK). Briefly, bacterial genomic DNA was extracted using ZymoBIOMICS DNA Kits (Zymo Research, Orange, CA, USA) and quantified in triplicates with the Quantit dsDNA HS assay in an Ependorff AF2200 plate reader. Genomic DNA libraries were prepared using a Nextera XT Library Prep Kit (Illumina, San Diego, CA, USA), following the manufacturer’s protocol. Pooled libraries were quantified using the Kapa Biosystems Library Quantification Kit for Illumina on a Roche light cycler 96 qPCR machine. Libraries were sequenced on an Illumina instrument using a 250 bp paired end protocol.

### 4.5. Bioinformatics Analysis

Illumina short-reads were adapter-trimmed using Trimmomatic 0.30 with a sliding window quality cutoff of Q15 [40]. De novo assemblies were performed using SPAdes version 3.7 (Columbia, SC, USA) [41], and contigs were annotated using Prokka 1.11 (Carlton, Australia) [42]. Antimicrobial-resistant genes were detected using ResFinder 4.1 (Center for Genomic Epidemiology, Lyngby, Denmark) [43] and the Comprehensive Antibiotic Resistance Database (CARD 2.0) (Hamilton, ON, Canada) [44]. Plasmid replicons were analyzed using PlasmidFinder 2.1 (Center for Genomic Epidemiology, Lyngby, Denmark) [45] and PLACNETw (Universidad de Cantabria & Instituto de Biomedicinay Biotecnología de Cantabria, Santander, Spain) [46]. Default parameters were used for all software programs unless otherwise specified. 

To investigate penicillin non-susceptibility, we analyzed the predicted translated sequences of five *pbp* genes (*pbp1a, pbp1b, pbp2a, pbp2b,* and pbp2x) using local BLAST + and Clustal W. *S. suis* P1/7 (Genbank accession number NC_009648.1 (accessed on 21 June 2021)) was used as the penicillin-susceptible reference strain. A neighbor-joining phylogenetic tree was constructed using MEGA X with 1000 bootstrap replicates by applying the Dayhoff model [47]. The tree was visualized and annotated using Interactive Tree of Life (iTOL) v5 (Heidelberg, Germany) [48].

To search for the genetically closest relatives to the 10 selected penicillin-non-susceptible isolates, a modular single genome analysis following the core genome multilocus sequence typing (cgMLST) approach by BacWGSTdb 2.0 was used (Institute of Translational Medicine, Zhejiang University, Hangzhou, China) [49]. The genetically closest relatives were chosen for 5–10 strains based on small numbers of allelic differences with selection thresholds of 100 to 500, depending on the isolate under study. The genomic comparisons of 10 penicillin-non-susceptible isolates and the closest relatives selected from BacWGSTdb were conducted using a reference genome-based single-nucleotide polymorphism (SNP) strategy with CSI phylogeny (Center for Genomic Epidemiology, Lyngby, Denmark) [50]. Phylogenetic trees were built using MEGA X via the neighbor-joining method with 1000 bootstrap replicates by applying the Tamura three-parameter model [47]. The phylogenetic tree was visualized using the iTOL v5 [48]. *S. suis* S735, a type strain (accession no. CP003736 (accessed on 21 June 2021)), was used as the reference sequences for SNP analysis. 

### 4.6. Accession Numbers

The genome sequences of the 10 non-penicillin-susceptible *S. suis* isolates were deposited in the NCBI GenBank under Bioproject accession number PRJNA691075 with the following strains: ID40747 (JAFEIV000000000), ID40085 (JAFEIX000000000), ID40484 (JAFEIY000000000), ID37647 (JAFEIZ000000000), ID30190 (JAFEIT000000000), ID32098 (JAFEIR000000000), ID32494 (JAFEIS000000000), ID27715 (JAFEIU000000000), ID41126 (JAFEIW000000000), and ID33329 (CP068708).

## 5. Conclusions

We have presented the characteristics of antimicrobial resistance of *S. suis* isolates recovered from humans. Resistance to tetracycline, macrolide, and clindamycin with *tet* (O) and *ermB* as the predominant resistance genes was commonly detected among isolates. Non-susceptibility to penicillin was shown in 8.3% of isolates, especially serotype 2-ST233 as the predominant group. In the “from farm to table” model, antimicrobial resistance is developed through the usage of antimicrobial agents on farms, and spread from animals to people via animal product consumption, direct contact with the animals, or through the environment. The “One Health” approach is a useful tool to combat AMR in bacteria by coordinating the human, animal, and environmental sectors. In addition, improvements in food hygiene standards and biosecurity measures at farms and in slaughtering procedures using hazard analysis and critical control point (HACCP) criteria are recommended, as well as better standards of hygiene in retail markets. Hence, restrictions on antimicrobial use, and the continuous control, monitoring, and surveillance of *S. suis* throughout the pork supply chain are top priority concerns and crucial for ensuring good public health outcomes.

## Figures and Tables

**Table 1:** Minimal inhibitory concentrations of penicillin against intermediately resistant *S. suis* isolates.

Serotypes	CC	ST	MIC (µg/mL)	No. of Isolates.
2	1	1	0.38	2
	25	25	0.38	1
	104	104	0.25	3
	1	105	0.38	1
	233/379	233	0.25	1
			0.38	7
			0.5	9
			0.75	3
			1	1
			1.5	1
		379	0.25	1
			0.5	1
			0.75	1
	104	393	0.5	1
5	-	235	1	1
14	1	105	0.5	1
24	221/234	221	0.75	1
		234	0.38	1
Total				37

CC: Clonal complex; ST: Sequence type.

**Table 2:** Distribution of macrolide- and tetracycline-resistance genes in *S. suis*.

Serotype	No. of Isolates	Macrolide-Resistance Genes				Tetracycline-Resistance Genes					
		*ermB*	*mefA*	*msrD*	Unknown	*tetO*	*tetM*	*tetM+ tetL*	*tetW*	*tetO+ tetW*	Unknown
1	2 (0.45%)	2 (0.45%)	-	-	-	2 (0.45%)	0	0	0	0	0
2	392 (87.50%)	334 (74.55%)	-	-	58 (12.95%)	357 (79.69%)	1 (0.22%)	1 (0.22%)	-	26 (5.80%)	7 (1.56%)
4	1 (0.22%)	1 (0.22%)	-	-	-	1 (0.22%)	0	0	0	0	0
5	2 (0.45%)	2 (0.45%)	-	-	-	2 (0.45%)	0	0	0	0	0
9	1 (0.22%)	-	-	-	1 (0.22%)-	0	0	0	1 (0.22%)	0	0
14	47 (10.49%)	42 (9.37%)	-	-	5 (1.12%)	44 (9.82%)	-	1 (0.22%)	-	-	2 (0.45%)
24	3 (0.67%)	3 (0.67%)	-	-	-	3 (0.67%)	0	0	0	0	0
Total	448 (100%)	384 (85.71%)	0	0	64 (14.29%)	409 (91.29%)	1 (0.22%)	2 (0.45%)	1 (0.22%)	26 (5.8%)	9 (2.0%)

**Table 3:** Whole-genome analysis of 10 non-penicillin-susceptible *S. suis* isolates isolated from humans.

Serotype	CC	ST	Strain ID No.	Plasmid Replicon Types	Acquired Antimicrobial-Resistance Genes			Substitutions in *pbp* Amino Acid				
					ML	TET	AMG	*pbp1a*	*pbp1b*	*pbp2a*	*pbp2b*	*pbp2x*
2	25	25	30190	MOBV, MOBT	*ermB*	*tetO*	-	T249P, A365S	G18V, I267V, K372G, A419S, V492I, T578A A765P	H27R, I121V, N233D, A275T, A561S, S660T	I414V, V637M, V641I	I72T, D136A, L204P, A468S,
	104	104	27715	MOBP, MOBT	*ermB, InuB, IsaE*	*tetO*	*aac(6′)-aph(2″), aph(3′)-III*	E447A, A493T, T689A, A703S	G18V, I267V, K372G, A419S, G476R, T578A A765P	H19N, R22Q, H27R, I34V, I121V, N233D V390I, A434S, A561S, S637N, S660T	K143E, I414V, V641I, A659V	I72T, L109F, K110Q, K111E, V112I, Q116E, S121H, E125D, F127L, K128N, H130Y, M139N, E140K, E146D, S165T, E177K, V205I, V217M, L220M, V245I, K251S, T254Q, T276I, N284T, R288K, Q321E, Y382Q, Y389F, Q407E, T418A, F422Y, S450T, I474V, V494I, S495A, S496A, Y525F, L533V, D541E, V547M, T551S, S556G, Q560L, G565D, I568T, N595S, D601T, S621V, P624S, A625N, A627S, R628L, V629L, A630T, Q636K, L640I, I641V, S648I, Q655N, A666S, K670Q, V671A, S677T, K678A, I680S, I701V, I708L, F709I, K720E, T722Q, S724A, V727I, A736K, K742N, I745V
	233/379	233	40747	MOBP, MOBT	*ermB*	*tetO*	-	E447A, A493T, T689A, A703S	G18V, I267V, K372G, A419S, G476R, T578A A765P	H19N, R22Q, H27R, I34V, I121V, N233D V390I, A434S, A561S, S637N, S660T	P92S, K143E, N179S, S206A, I300V, R332K N356E, S374A, S375G, T376S, I414V, Y432W I452A, K479T, T507I, D512E, K513E, T515S D587E, T625R, K674N	I72T, L109F, K110Q, K111E, V112I, Q116E, S121H, E125D, F127L, K128N, H130Y, M139N, E140K, E146D, S165T, E177K, V205I, V217M, L220M, V245I, K251S, T254Q, N284T, R288K, Q321E, Y382Q, Y389F, Q407E, T418A, F422Y, S450T, I474V, V494I, S495A, S496A, Y525F, L533V, D541E, V547M, T551S, S556G, Q560L, G565D, I568T, R628Q, K678N, K741E
			41126	MOBP, MOBT	*ermB*	*tetO*	-	E447A, A493T, T689A, A703S	G18V, I267V, K372G, A419S, G476R, T578A A765P	H19N, R22Q, H27R, I34V, I121V, N233D V390I, A434S, A561S, S637N, S660T	P92S, K143E, N179S, S206A, I300V, R332K N356E, S374A, S375G, T376S, I414V, Y432W I452A, K479T, T507I, D512E, K513E, T515S D587E, T625R, K674N	V18I, I72T, E146K, N182D, P190S, L204P, H212Y, S450T, D486N, T551S, I568T, R628Q, K678N, K741E
			40484	MOBP, MOBT	*ermB*	*tetO*	-	E447A, A493T, T689A, A703S	G18V, I267V, K372G, A419S, G476R, T578A A765P	H19N, R22Q, H27R, I34V, I121V, N233D V390I, A434S, A561S, S637N, S660T	P92S, K143E, N179S, S206A, I300V, R332K N356E, S374A, S375G, T376S, I414V, Y432W I452A, K479T, T507I, D512E, K513E, T515S D587E, T625R, K674N	I72T, L109F, K110Q, K111E, V112I, Q116E, S121H, E125D, F127L, K128N, H130Y, M139N, E140K, E146D, S165T, E177K, V205I, V217M, L220M, V245I, K251S, T254Q, N284T, R288K, Q321E, Y382Q, Y389F, Q407E, T418A, F422Y, S450T, I474V, V494I, S495A, S496A, Y525F, L533V, D541E, V547M, T551S, S556G, Q560L, G565D, I568T, R628Q, K678N, K741E
			37647	MOBP, MOBT	*ermB*	*tetO*	-	E447A, A493T, T689A, A703S	G18V, I267V, K372G, A419S, G476R, T578A A765P	H19N, R22Q, H27R, I34V, I121V, N233D V390I, A434S, A561S, S637N, S660T	P92S, K143E, N179S, S206A, I300V, R332K N356E, S374A, S375G, T376S, I414V, Y432W I452A, K479T, T507I, D512E, K513E, T515S D587E, T625R, K674N	V18I, I72T, E146K, N182D, P190S, L204P, H212Y, S450T, D486N, T551S, I568T, R628Q, K678N, K741E
		379	40085	MOBP, MOBT	*ermB*	*tetO*	-	E447A, A493T, T689A, A703S	G18V, I267V, K372G, A419S, G476R, T578A A765P	H19N, R22Q, H27R, I34V, I121V, N233D V390I, A434S, A561S, S637N, S660T	P92S, K143E, N179S, S206A, I300V, R332K N356E, S374A, S375G, T376S, I414V, Y432W I452A, K479T, T507I, D512E, K513E, T515S D587E, T625R, K674N	V18I, I72T, E146K, N182D, P190S, L204P, H212Y, S450T, D486N, T551S, I568T, R628Q, K678N, K741E
14	1	105	32494	Rep21, MOBT	*ermB, InuA*	*tetO*	-	-	-	-	-	-
24	221/234	221	33329	MOBP, MOBV	*ermB*	*tetO*	-	A231V, A289E, P409T, V412F, N459D, H464Y S477G, K479Q, K522Q, K525Q, N550D, S578A S675Y, T689A, A703S	S259A, K317E, K338E, K372G, T578A, A765P S779T	H27R, I34V, I121V, N233D, R379T, S430A M433T, A434P, L479F, Y487F, V504A, A522S T523A, K527D, E549Q, A561S, A568S, T584A A585T, T578L, T581S, H588Y, I589L, H610Y S638G, S644T, Q647R, N651D, S652A, Y656N S660T, K661S, E663D, A669V, E698D	P92S, K143E, N179S, S206A, T231N, I240V S266P, Q291E, I300V, E304K, R332K, A340S V408I, I414V, Q415L, Y432W, I452V, K479T D512E, K513E, T515S, T521M, A539V, I542V T547K, A562P, E568D, E586D, D587E, V598L S602G, A633P, D636E, Q668E, S669N, T675A Q678T, Q685N, H688Y	I72T, D136A, A195S, L204P, T418A, A468S, T551S, I568T, D579N, N595S, D601S, V603I, S621A, A625S, A627T, R628Y, A630E, N631K, E634D, Q636K, K678N
		234	32098	MOBP, MOBV	*ermB*	*tetO*	-	A231V, A289E, P409T, V412F, N459D, H464Y S477G, K479Q, K522Q, K525Q, N550D, S578A S675Y, T689A, A703S	S259A, K317E, K338E, K372G, T578A, A765P S779T	H27R, I34V, I121V, N233D, R379T, S430A M433T, A434P, L479F, Y487F, V504A, A522S T523A, K527D, E549Q, A561S, A568S, T584A A585T, T578L, T581S, H588Y, I589L, H610Y S638G, S644T, Q647R, N651D, S652A, Y656N S660T, K661S, E663D, A669V, E698D	P92S, K143E, N179S, S206A, T231N, I240V S266P, Q291E, I300V, E304K, R332K, A340S V408I, I414V, Q415L, Y432W, I452V, K479T D512E, K513E, T515S, T521M, A539V, I542V T547K, A562P, E568D, E586D, D587E, V598L S602G, A633P, D636E, Q668E, S669N, T675A Q678T, Q685N, H688Y	I72T, D136A, A195S, L204P, T418A, A468S, T551S, I568T, D579N, N595S, D601S, V603I, S621A, A625S, A627T, R628Y, A630E, N631K, E634D, Q636K, K678N

CC: clonal complex; ST: sequence type; ML: macrolide; TET: tetracycline; AMG: aminoglycoside. Substitutions of *pbp* amino acid were determined using *S. suis* P1/7 as a reference penicillin-susceptible strain.

## Data Availability

No new data were created or analyzed in this study. Data sharing is not applicable to this article.

## Supplementary Materials

The following are available online at https://www.mdpi.com/article/10.3390/pathogens10091178/s1. Table S1. Antimicrobial resistance patterns and the resistance genes of *S. suis* by serotypes and sequence types, Table S2. Detail of *Streptococcus suis* used in this study, Table S3. PCR primers for the detection of tetracycline- and macrolide-resistant genes, Supplemental file 1. Alignment of the pbp1A sequence, Supplemental file 2. Alignment of the pbp1B sequence, Supplemental file 3. Alignment of the pbp2A sequence, Supplemental file 4. Alignment of the pbp2B sequence, Supplemental file 5. Alignment of the pbp2X sequence.
