# Supplementary material for: Non-Penicillin-Susceptible Streptococcus suis Isolated from Humans
Source: Pathogens. 2021 Sep 13;10(9):1178. doi: 10.3390/pathogens10091178 (PMC8471365; doi:10.3390/pathogens10091178)
Supplement: Supplementary file 1 [file pathogens-10-01178-s001.zip › Supplementary file 1_pbp1a alignment result.pdf]

**Alignment of pbp1A sequence**

```
pbp1a_32098      MVNLLKTTTIKKALLITANILLAGAIIACLAGAALVVYYIQSAPELTEESLTATVSSKIY    60
pbp1a_33329      MVNLLKTTTIKKALLITANILLAGAIIACLAGAALVVYYIQSAPELTEESLTATVSSKIY    60
pbp1a_22715      MVNLLKTTTIKKALLITANILLAGAIIACLAGAALVVYYIQSAPELTEESLTATVSSKIY    60
pbp1a_37647      MVNLLKTTTIKKALLITANILLAGAIIACLAGAALVVYYIQSAPELTEESLTATVSSKIY    60
pbp1a_40085      MVNLLKTTTIKKALLITANILLAGAIIACLAGAALVVYYIQSAPELTEESLTATVSSKIY    60
pbp1a_40484      MVNLLKTTTIKKALLITANILLAGAIIACLAGAALVVYYIQSAPELTEESLTATVSSKIY    60
pbp1a_40747      MVNLLKTTTIKKALLITANILLAGAIIACLAGAALVVYYIQSAPELTEESLTATVSSKIY    60
pbp1a_41126      MVNLLKTTTIKKALLITANILLAGAIIACLAGAALVVYYIQSAPELTEESLTATVSSKIY    60
pbp1a_30190      MVNLLKTTTIKKALLITANILLAGAIIACLAGAALVVYYIQSAPELTEESLTATVSSKIY    60
pbp1a_32494      MVNLLKTTTIKKALLITANILLAGAIIACLAGAALVVYYIQSAPELTEESLTATVSSKIY    60
pbp1a_P1/7       MVNLLKTTTIKKALLITANILLAGAIIACLAGAALVVYYIQSAPELTEESLTATVSSKIY    60
                 ************************************************************

pbp1a_32098      DKNGNLIADLGAEKRSSAKTEEIPTDLVNAIVAIEDQRFFNHRGVDVIRIAGSLINNLSG    120
pbp1a_33329      DKNGNLIADLGAEKRSSAKTEEIPTDLVNAIVAIEDQRFFNHRGVDVIRIAGSLINNLSG    120
pbp1a_22715      DKNGNLIADLGAEKRSSAKTEEIPTDLVNAIVAIEDQRFFNHRGVDVIRIAGSLINNLSG    120
pbp1a_37647      DKNGNLIADLGAEKRSSAKTEEIPTDLVNAIVAIEDQRFFNHRGVDVIRIAGSLINNLSG    120
pbp1a_40085      DKNGNLIADLGAEKRSSAKTEEIPTDLVNAIVAIEDQRFFNHRGVDVIRIAGSLINNLSG    120
pbp1a_40484      DKNGNLIADLGAEKRSSAKTEEIPTDLVNAIVAIEDQRFFNHRGVDVIRIAGSLINNLSG    120
pbp1a_40747      DKNGNLIADLGAEKRSSAKTEEIPTDLVNAIVAIEDQRFFNHRGVDVIRIAGSLINNLSG    120
pbp1a_41126      DKNGNLIADLGAEKRSSAKTEEIPTDLVNAIVAIEDQRFFNHRGVDVIRIAGSLINNLSG    120
pbp1a_30190      DKNGNLIADLGAEKRSSAKTEEIPTDLVNAIVAIEDQRFFNHRGVDVIRIAGSLINNLSG    120
pbp1a_32494      DKNGNLIADLGAEKRSSAKTEEIPTDLVNAIVAIEDQRFFNHRGVDVIRIAGSLINNLSG    120
pbp1a_P1/7       DKNGNLIADLGAEKRSSAKTEEIPTDLVNAIVAIEDQRFFNHRGVDVIRIAGSLINNLSG    120
                 ************************************************************

pbp1a_32098      GRLQGGSTLDQQFIKLTYFSTSVEDQNLKRKIQEAWLATQLERRNTKQEILTYYINKVYM    180
pbp1a_33329      GRLQGGSTLDQQFIKLTYFSTSVEDQNLKRKIQEAWLATQLERRNTKQEILTYYINKVYM    180
pbp1a_22715      GRLQGGSTLDQQFIKLTYFSTSVEDQNLKRKIQEAWLATQLERRNTKQEILTYYINKVYM    180
pbp1a_37647      GRLQGGSTLDQQFIKLTYFSTSVEDQNLKRKIQEAWLATQLERRNTKQEILTYYINKVYM    180
pbp1a_40085      GRLQGGSTLDQQFIKLTYFSTSVEDQNLKRKIQEAWLATQLERRNTKQEILTYYINKVYM    180
pbp1a_40484      GRLQGGSTLDQQFIKLTYFSTSVEDQNLKRKIQEAWLATQLERRNTKQEILTYYINKVYM    180
pbp1a_40747      GRLQGGSTLDQQFIKLTYFSTSVEDQNLKRKIQEAWLATQLERRNTKQEILTYYINKVYM    180
pbp1a_41126      GRLQGGSTLDQQFIKLTYFSTSVEDQNLKRKIQEAWLATQLERRNTKQEILTYYINKVYM    180
pbp1a_30190      GRLQGGSTLDQQFIKLTYFSTSVEDQNLKRKIQEAWLATQLERRNTKQEILTYYINKVYM    180
pbp1a_32494      GRLQGGSTLDQQFIKLTYFSTSVEDQNLKRKIQEAWLATQLERRNTKQEILTYYINKVYM    180
pbp1a_P1/7       GRLQGGSTLDQQFIKLTYFSTSVEDQNLKRKIQEAWLATQLERRNTKQEILTYYINKVYM    180
                 ************************************************************

pbp1a_32098      SNGNYGMKTAALAFYGKELKDLTLPQLALLAGMPQAPNQYDPYTNPEDAKVRRDLVLAEM    240
pbp1a_33329      SNGNYGMKTAALAFYGKELKDLTLPQLALLAGMPQAPNQYDPYTNPEDAKVRRDLVLAEM    240
pbp1a_22715      SNGNYGMKTAALAFYGKELKDLTLPQLALLAGMPQAPNQYDPYTNPEDAKARRDLVLAEM    240
pbp1a_37647      SNGNYGMKTAALAFYGKELKDLTLPQLALLAGMPQAPNQYDPYTNPEDAKARRDLVLAEM    240
pbp1a_40085      SNGNYGMKTAALAFYGKELKDLTLPQLALLAGMPQAPNQYDPYTNPEDAKARRDLVLAEM    240
pbp1a_40484      SNGNYGMKTAALAFYGKELKDLTLPQLALLAGMPQAPNQYDPYTNPEDAKARRDLVLAEM    240
pbp1a_40747      SNGNYGMKTAALAFYGKELKDLTLPQLALLAGMPQAPNQYDPYTNPEDAKARRDLVLAEM    240
pbp1a_41126      SNGNYGMKTAALAFYGKELKDLTLPQLALLAGMPQAPNQYDPYTNPEDAKARRDLVLAEM    240
pbp1a_30190      SNGNYGMKTAALAFYGKELKDLTLPQLALLAGMPQAPNQYDPYTNPEDAKARRDLVLAEM    240
pbp1a_32494      SNGNYGMKTAALAFYGKELKDLTLPQLALLAGMPQAPNQYDPYTNPEDAKARRDLVLAEM    240
pbp1a_P1/7       SNGNYGMKTAALAFYGKELKDLTLPQLALLAGMPQAPNQYDPYTNPEDAKARRDLVLAEM    240
                 **************************************************.*********

pbp1a_32098      LEEKYIDNTQYEQAVLTPVTDGLQPLSNAAAYPAYMDNYLKQVVEEVEEKTGYNLLTTGM    300
pbp1a_33329      LEEKYIDNTQYEQAVLTPVTDGLQPLSNAAAYPAYMDNYLKQVVEEVEEKTGYNLLTTGM    300
pbp1a_22715      LEEKYIDNTQYEQAVLTPVTDGLQPLSNAAAYPAYMDNYLKQVVEEVEAKTGYNLLTTGM    300
pbp1a_37647      LEEKYIDNTQYEQAVLTPVTDGLQPLSNAAAYPAYMDNYLKQVVEEVEAKTGYNLLTTGM    300
pbp1a_40085      LEEKYIDNTQYEQAVLTPVTDGLQPLSNAAAYPAYMDNYLKQVVEEVEAKTGYNLLTTGM    300
pbp1a_40484      LEEKYIDNTQYEQAVLTPVTDGLQPLSNAAAYPAYMDNYLKQVVEEVEAKTGYNLLTTGM    300
pbp1a_40747      LEEKYIDNTQYEQAVLTPVTDGLQPLSNAAAYPAYMDNYLKQVVEEVEAKTGYNLLTTGM    300
pbp1a_41126      LEEKYIDNTQYEQAVLTPVTDGLQPLSNAAAYPAYMDNYLKQVVEEVEAKTGYNLLTTGM    300
pbp1a_30190      LEEKYIDNPQYEQAVLTPVTDGLQPLSNAAAYPAYMDNYLKQVVEEVEAKTGYNLLTTGM    300
pbp1a_32494      LEEKYIDNTQYEQAVLTPVTDGLQPLSNAAAYPAYMDNYLKQVVEEVEAKTGYNLLTTGM    300
pbp1a_P1/7       LEEKYIDNTQYEQAVLTPVTDGLQPLSNAAAYPAYMDNYLKQVVEEVEAKTGYNLLTTGM    300
                 ******** *************************************** ***********
```

```
pbp1a_32098      DVYTNVDPAAQQQLWNIYNTDMYVSYPDDLLQVASTVVDVSNGKVVAQLGGRKQETNVSF      360
pbp1a_33329      DVYTNVDPAAQQQLWNIYNTDMYVSYPDDLLQVASTVVDVSNGKVVAQLGGRKQETNVSF      360
pbp1a_22715      DVYTNVDPAAQQQLWNIYNTDMYVSYPDDLLQVASTVVDVSNGKVVAQLGGRKQETNVSF      360
pbp1a_37647      DVYTNVDPAAQQQLWNIYNTDMYVSYPDDLLQVASTVVDVSNGKVVAQLGGRKQETNVSF      360
pbp1a_40085      DVYTNVDPAAQQQLWNIYNTDMYVSYPDDLLQVASTVVDVSNGKVVAQLGGRKQETNVSF      360
pbp1a_40484      DVYTNVDPAAQQQLWNIYNTDMYVSYPDDLLQVASTVVDVSNGKVVAQLGGRKQETNVSF      360
pbp1a_40747      DVYTNVDPAAQQQLWNIYNTDMYVSYPDDLLQVASTVVDVSNGKVVAQLGGRKQETNVSF      360
pbp1a_41126      DVYTNVDPAAQQQLWNIYNTDMYVSYPDDLLQVASTVVDVSNGKVVAQLGGRKQETNVSF      360
pbp1a_30190      DVYTNVDPAAQQQLWNIYNTDMYVSYPDDLLQVASTVVDVSNGKVVAQLGGRKQETNVSF      360
pbp1a_32494      DVYTNVDPAAQQQLWNIYNTDMYVSYPDDLLQVASTVVDVSNGKVVAQLGGRKQETNVSF      360
pbp1a_P1/7       DVYTNVDPAAQQQLWNIYNTDMYVSYPDDLLQVASTVVDVSNGKVVAQLGGRKQETNVSF      360
                 ************************************************************

pbp1a_32098      GTNQAVETNRDFGSTMKPITDYAPAFENGIYTSTADLILDGPYNYPGTTTPFNNWDKQYY      420
pbp1a_33329      GTNQAVETNRDFGSTMKPITDYAPAFENGIYTSTADLILDGPYNYPGTTTPFNNWDKQYY      420
pbp1a_22715      GTNQAVETNRDFGSTMKPITDYAPAFENGIYTSTADLILDGPYNYPGTPTPVNNWDKQYY      420
pbp1a_37647      GTNQAVETNRDFGSTMKPITDYAPAFENGIYTSTADLILDGPYNYPGTPTPVNNWDKQYY      420
pbp1a_40085      GTNQAVETNRDFGSTMKPITDYAPAFENGIYTSTADLILDGPYNYPGTPTPVNNWDKQYY      420
pbp1a_40484      GTNQAVETNRDFGSTMKPITDYAPAFENGIYTSTADLILDGPYNYPGTPTPVNNWDKQYY      420
pbp1a_40747      GTNQAVETNRDFGSTMKPITDYAPAFENGIYTSTADLILDGPYNYPGTPTPVNNWDKQYY      420
pbp1a_41126      GTNQAVETNRDFGSTMKPITDYAPAFENGIYTSTADLILDGPYNYPGTPTPVNNWDKQYY      420
pbp1a_30190      GTNQSVETNRDFGSTMKPITDYAPAFENGIYTSTADLILDGPYNYPGTPTPVNNWDKQYY      420
pbp1a_32494      GTNQAVETNRDFGSTMKPITDYAPAFENGIYTSTADLILDGPYNYPGTPTPVNNWDKQYY      420
pbp1a_P1/7       GTNQAVETNRDFGSTMKPITDYAPAFENGIYTSTADLILDGPYNYPGTPTPVNNWDKQYY      420
                 ****:******************************************* ** .********

pbp1a_32098      GNISVKTAIQYSRNVTAVKALEATGLENALKFLNSVGIDYPDIYYSNAISSNTSDTGSQY      480
pbp1a_33329      GNISVKTAIQYSRNVTAVKALEATGLENALKFLNSVGIDYPDIYYSNAISSNTSDTGSQY      480
pbp1a_22715      GNISVKTAIQYSRNVTAVKALEATGLANALKFLNSVGINYPDIHYSNAISSNTSDTSSKY      480
pbp1a_37647      GNISVKTAIQYSRNVTAVKALEATGLANALKFLNSVGINYPDIHYSNAISSNTSDTSSKY      480
pbp1a_40085      GNISVKTAIQYSRNVTAVKALEATGLANALKFLNSVGINYPDIHYSNAISSNTSDTSSKY      480
pbp1a_40484      GNISVKTAIQYSRNVTAVKALEATGLANALKFLNSVGINYPDIHYSNAISSNTSDTSSKY      480
pbp1a_40747      GNISVKTAIQYSRNVTAVKALEATGLANALKFLNSVGINYPDIHYSNAISSNTSDTSSKY      480
pbp1a_41126      GNISVKTAIQYSRNVTAVKALEATGLANALKFLNSVGINYPDIHYSNAISSNTSDTSSKY      480
pbp1a_30190      GNISVKTAIQYSRNVTAVKALEATGLENALKFLNSVGINYPDIHYSNAISSNTSDTSSKY      480
pbp1a_32494      GNISVKTAIQYSRNVTAVKALEATGLENALKFLNSVGINYPDIHYSNAISSNTSDTSSKY      480
pbp1a_P1/7       GNISVKTAIQYSRNVTAVKALEATGLENALKFLNSVGINYPDIHYSNAISSNTSDTSSKY      480
                 ************************** ***********:****:************.*:*

pbp1a_32098      GASSEKMAAAYAAFANGGTYYAPQYVNKIVFSDGTVTEYAPQGTQVMSAETAYMMTDMMK      540
pbp1a_33329      GASSEKMAAAYAAFANGGTYYAPQYVNKIVFSDGTVTEYAPQGTQVMSAETAYMMTDMMK      540
pbp1a_22715      GASSEKMAAAYATFANGGTYYAPQYVNKIVFSDGTVTEYAPKGTKVMSAETAYMMTDMMK      540
pbp1a_37647      GASSEKMAAAYATFANGGTYYAPQYVNKIVFSDGTVTEYAPKGTKVMSAETAYMMTDMMK      540
pbp1a_40085      GASSEKMAAAYATFANGGTYYAPQYVNKIVFSDGTVTEYAPKGTKVMSAETAYMMTDMMK      540
pbp1a_40484      GASSEKMAAAYATFANGGTYYAPQYVNKIVFSDGTVTEYAPKGTKVMSAETAYMMTDMMK      540
pbp1a_40747      GASSEKMAAAYATFANGGTYYAPQYVNKIVFSDGTVTEYAPKGTKVMSAETAYMMTDMMK      540
pbp1a_41126      GASSEKMAAAYATFANGGTYYAPQYVNKIVFSDGTVTEYAPKGTKVMSAETAYMMTDMMK      540
pbp1a_30190      GASSEKMAAAYAAFANGGTYYAPQYVNKIVFSDGTVTEYAPKGTKVMSAETAYMMTDMMK      540
pbp1a_32494      GASSEKMAAAYAAFANGGTYYAPQYVNKIVFSDGTVTEYAPKGTKVMSAETAYMMTDMMK      540
pbp1a_P1/7       GASSEKMAAAYAAFANGGTYYAPQYVNKIVFSDGTVTEYAPKGTKVMSAETAYMMTDMMK      540
                 ************:****************************:**:***************

pbp1a_32098      AVMSYGYGLDASVSGVPMAGKTGTSNYTDSETDTILAAIPEANYSYMVVPDENFVGYSSQ      600
pbp1a_33329      AVMSYGYGLDASVSGVPMAGKTGTSNYTDSETDTILAAIPEANYSYMVVPDENFVGYSSQ      600
pbp1a_22715      AVMSYGYGLNASVSGVPMAGKTGTSNYTDSETDTILASIPEANYSYMVVPDENFVGYSSQ      600
pbp1a_37647      AVMSYGYGLNASVSGVPMAGKTGTSNYTDSETDTILASIPEANYSYMVVPDENFVGYSSQ      600
pbp1a_40085      AVMSYGYGLNASVSGVPMAGKTGTSNYTDSETDTILASIPEANYSYMVVPDENFVGYSSQ      600
pbp1a_40484      AVMSYGYGLNASVSGVPMAGKTGTSNYTDSETDTILASIPEANYSYMVVPDENFVGYSSQ      600
pbp1a_40747      AVMSYGYGLNASVSGVPMAGKTGTSNYTDSETDTILASIPEANYSYMVVPDENFVGYSSQ      600
pbp1a_41126      AVMSYGYGLNASVSGVPMAGKTGTSNYTDSETDTILASIPEANYSYMVVPDENFVGYSSQ      600
pbp1a_30190      AVMSYGYGLNASVSGVPMAGKTGTSNYTDSETDTILASIPEANYSYMVVPDENFVGYSSQ      600
pbp1a_32494      AVMSYGYGLNASVSGVPMAGKTGTSNYTDSETDTILASIPEANYSYMVVPDENFVGYSSQ      600
pbp1a_P1/7       AVMSYGYGLNASVSGVPMAGKTGTSNYTDSETDTILASIPEANYSYMVVPDENFVGYSSQ      600
                 *********:***************************:**********************
```

```
pbp1a_32098      YAMAVWTGYTNRMTPILDNSMRIATDVYHNMMLFMHSDYTATDWEMPSGLVRYGSNYYLR      660
pbp1a_33329      YAMAVWTGYTNRMTPILDNSMRIATDVYHNMMLFMHSDYTATDWEMPSGLVRYGSNYYLR      660
pbp1a_22715      YAMAVWTGYTNRMTPILDNSMRIATDVYHNMMLFMHSDYTATDWEMPSGLVRYGSNYYLR      660
pbp1a_37647      YAMAVWTGYTNRMTPILDNSMRIATDVYHNMMLFMHSDYTATDWEMPSGLVRYGSNYYLR      660
pbp1a_40085      YAMAVWTGYTNRMTPILDNSMRIATDVYHNMMLFMHSDYTATDWEMPSGLVRYGSNYYLR      660
pbp1a_40484      YAMAVWTGYTNRMTPILDNSMRIATDVYHNMMLFMHSDYTATDWEMPSGLVRYGSNYYLR      660
pbp1a_40747      YAMAVWTGYTNRMTPILDNSMRIATDVYHNMMLFMHSDYTATDWEMPSGLVRYGSNYYLR      660
pbp1a_41126      YAMAVWTGYTNRMTPILDNSMRIATDVYHNMMLFMHSDYTATDWEMPSGLVRYGSNYYLR      660
pbp1a_30190      YAMAVWTGYTNRMTPILDNSMRIATDVYHNMMLFMHSDYTATDWEMPSGLVRYGSNYYLR      660
pbp1a_32494      YAMAVWTGYTNRMTPILDNSMRIATDVYHNMMLFMHSDYTATDWEMPSGLVRYGSNYYLR      660
pbp1a_P1/7       YAMAVWTGYTNRMTPILDNSMRIATDVYHNMMLFMHSDYTATDWEMPSGLVRYGSNYYLR      660
                 ************************************************************

pbp1a_32098      GSRSLSNAYNTYTSYSTSSSSSTETSETAEATTTSSETSSGTSTETATSPTATSSTETAT      720
pbp1a_33329      GSRSLSNAYNTYTSYSTSSSSSTETSETAEATTTSSETSSGTSTETATSPTATSSTETAT      720
pbp1a_22715      GSRSLSNAYNTYTSSSTSSSSSTETSETAEATTTSSETSSGTSTETAT-----SSTETAT      715
pbp1a_37647      GSRSLSNAYNTYTSSSTSSSSSTETSETAEATTTSSETSSGTSTETAT-----SSTETAT      715
pbp1a_40085      GSRSLSNAYNTYTSSSTSSSSSTETSETAEATTTSSETSSGTSTETAT-----SSTETAT      715
pbp1a_40484      GSRSLSNAYNTYTSSSTSSSSSTETSETAEATTTSSETSSGTSTETAT-----SSTETAT      715
pbp1a_40747      GSRSLSNAYNTYTSSSTSSSSSTETSETAEATTTSSETSSGTSTETAT-----SSTETAT      715
pbp1a_41126      GSRSLSNAYNTYTSSSTSSSSSTETSETAEATTTSSETSSGTSTETAT-----SSTETAT      715
pbp1a_30190      GSRSLSNAYNTYTSSSTSSSSSTETSETTEATTTSSETSSGTATETAT-----SSTETAT      715
pbp1a_32494      GSRSLSNAYNTYTSSSTSSSSSTETSETTEATTTSSETSSGTATETAT-----SSTETAT      715
pbp1a_P1/7       GSRSLSNAYNTYTSSSTSSSSSTETSETTEATTTSSETSSGTATETAT-----SSTETAT      715
                 ************** *************:*************:*****     *******

pbp1a_32098      TGNNPATGQTDGQ    733
pbp1a_33329      TGNNPATGQTDGQ    733
pbp1a_22715      TGNNPATGQTDGQ    728
pbp1a_37647      TGNNPATGQTDGQ    728
pbp1a_40085      TGNNPATGQTDGQ    728
pbp1a_40484      TGNNPATGQTDGQ    728
pbp1a_40747      TGNNPATGQTDGQ    728
pbp1a_41126      TGNNPATGQTDGQ    728
pbp1a_30190      TGNNPATGQTDGQ    728
pbp1a_32494      TGNNPATGQTDGQ    728
pbp1a_P1/7       TGNNPATGQTDGQ    728
                 *************
```
