# Supplementary material for: Non-Penicillin-Susceptible Streptococcus suis Isolated from Humans
Source: Pathogens. 2021 Sep 13;10(9):1178. doi: 10.3390/pathogens10091178 (PMC8471365; doi:10.3390/pathogens10091178)
Supplement: Supplementary file 1 [file pathogens-10-01178-s001.zip › Supplementary file 2_pbp1b alignment result.pdf]

Alignment of pbp1B sequence

```
pbp1b_32098      MATKSDKQDFKKKISALGLGDVVGVFLRTLKLLFNSVAVLVFLFGLFGAGIGIGFVVSLF     60
pbp1b_33329      MATKSDKQDFKKKISALGLGDVVGVFLRTLKLLFNSVAVLVFLFGLFGAGIGIGFVVSLF     60
pbp1B_P1/7       MATKSDKQDFKKKISALGLGDVVGVFLRTLKLLFNSVAVLVFLFGLFGAGIGIGFVVSLF     60
pbp1b_32494      MATKSDKQDFKKKISALGLGDVVGVFLRTLKLLFNSVAVLVFLFGLFGAGIGIGFVVSLF     60
pbp1b_22715      MATKSDKQDFKKKISALVLGDVVGVFLRTLKLLFNSVAVLVFLFGLFGAGIGIGFVVSLF     60
pbp1b_37647      MATKSDKQDFKKKISALVLGDVVGVFLRTLKLLFNSVAVLVFLFGLFGAGIGIGFVVSLF     60
pbp1b_40085      MATKSDKQDFKKKISALVLGDVVGVFLRTLKLLFNSVAVLVFLFGLFGAGIGIGFVVSLF     60
pbp1b_40484      MATKSDKQDFKKKISALVLGDVVGVFLRTLKLLFNSVAVLVFLFGLFGAGIGIGFVVSLF     60
pbp1b_40747      MATKSDKQDFKKKISALVLGDVVGVFLRTLKLLFNSVAVLVFLFGLFGAGIGIGFVVSLF     60
pbp1b_41126      MATKSDKQDFKKKISALVLGDVVGVFLRTLKLLFNSVAVLVFLFGLFGAGIGIGFVVSLF     60
pbp1b_30190      MATKSDKQDFKKKISALVLGDVVGVFLRTLKLLFNSVAVLVFLFGLFGAGIGIGFVVSLF     60
                 ***************** ******************************************

pbp1b_32098      DDVKIPKTEELVAKVSEVSRISTVTYSDGSLVSEVNSDLLRVPITSEEVSNYLKQAVIAT     120
pbp1b_33329      DDVKIPKTEELVAKVSEVSRISTVTYSDGSLVSEVNSDLLRVPITSEEVSNYLKQAVIAT     120
pbp1B_P1/7       DDVKIPKTEELVAKVSEVSRISTVTYSDGSLVSEVNSDLLRVPITSEEVSNYLKQAVIAT     120
pbp1b_32494      DDVKIPKTEELVAKVSEVSRISTVTYSDGSLVSEVNSDLLRVPITSEEVSNYLKQAVIAT     120
pbp1b_22715      DDVKIPKTEELVAKVSEVSRISTVTYSDGSLVSEVNSDLLRVPITSEEVSNYLKQAVIAT     120
pbp1b_37647      DDVKIPKTEELVAKVSEVSRISTVTYSDGSLVSEVNSDLLRVPITSEEVSNYLKQAVIAT     120
pbp1b_40085      DDVKIPKTEELVAKVSEVSRISTVTYSDGSLVSEVNSDLLRVPITSEEVSNYLKQAVIAT     120
pbp1b_40484      DDVKIPKTEELVAKVSEVSRISTVTYSDGSLVSEVNSDLLRVPITSEEVSNYLKQAVIAT     120
pbp1b_40747      DDVKIPKTEELVAKVSEVSRISTVTYSDGSLVSEVNSDLLRVPITSEEVSNYLKQAVIAT     120
pbp1b_41126      DDVKIPKTEELVAKVSEVSRISTVTYSDGSLVSEVNSDLLRVPITSEEVSNYLKQAVIAT     120
pbp1b_30190      DDVKIPKTEELVAKVSEVSRISTVTYSDGSLVSEVNSDLLRVPITSEEVSNYLKQAVIAT     120
                 ************************************************************

pbp1b_32098      EDETFETHNGVVPKAVLRAALGSVGLGSSSGGSTLTQQLIKQQLVGDAPTFTRKANEIVS     180
pbp1b_33329      EDETFETHNGVVPKAVLRAALGSVGLGSSSGGSTLTQQLIKQQLVGDAPTFTRKANEIVS     180
pbp1B_P1/7       EDETFETHNGVVPKAVLRAALGSVGLGSSSGGSTLTQQLIKQQLVGDAPTFTRKANEIVS     180
pbp1b_32494      EDETFETHNGVVPKAVLRAALGSVGLGSSSGGSTLTQQLIKQQLVGDAPTFTRKANEIVS     180
pbp1b_22715      EDETFETHNGVVPKAVLRAALGSVGLGSSSGGSTLTQQLIKQQLVGDAPTFTRKANEIVS     180
pbp1b_37647      EDETFETHNGVVPKAVLRAALGSVGLGSSSGGSTLTQQLIKQQLVGDAPTFTRKANEIVS     180
pbp1b_40085      EDETFETHNGVVPKAVLRAALGSVGLGSSSGGSTLTQQLIKQQLVGDAPTFTRKANEIVS     180
pbp1b_40484      EDETFETHNGVVPKAVLRAALGSVGLGSSSGGSTLTQQLIKQQLVGDAPTFTRKANEIVS     180
pbp1b_40747      EDETFETHNGVVPKAVLRAALGSVGLGSSSGGSTLTQQLIKQQLVGDAPTFTRKANEIVS     180
pbp1b_41126      EDETFETHNGVVPKAVLRAALGSVGLGSSSGGSTLTQQLIKQQLVGDAPTFTRKANEIVS     180
pbp1b_30190      EDETFETHNGVVPKAVLRAALGSVGLGSSSGGSTLTQQLIKQQLVGDAPTFTRKANEIVS     180
                 ************************************************************

pbp1b_32098      ALALERNMTKEEILTIYLNVSPFGRNNQGRNIAGVEAAAQGIFGKPAKDLTVPQAAFIAG     240
pbp1b_33329      ALALERNMTKEEILTIYLNVSPFGRNNQGRNIAGVEAAAQGIFGKPAKDLTVPQAAFIAG     240
pbp1B_P1/7       ALALERNMTKEEILTIYLNVSPFGRNNQGRNIAGVEAAAQGIFGKPAKDLTVPQAAFIAG     240
pbp1b_32494      ALALERNMTKEEILTIYLNVSPFGRNNQGRNIAGVEAAAQGIFGKPAKDLTVPQAAFIAG     240
pbp1b_22715      ALALERNMTKEEILTIYLNVSPFGRNNQGRNIAGVEAAAQGIFGKPAKDLTVPQAAFIAG     240
pbp1b_37647      ALALERNMTKEEILTIYLNVSPFGRNNQGRNIAGVEAAAQGIFGKPAKDLTVPQAAFIAG     240
pbp1b_40085      ALALERNMTKEEILTIYLNVSPFGRNNQGRNIAGVEAAAQGIFGKPAKDLTVPQAAFIAG     240
pbp1b_40484      ALALERNMTKEEILTIYLNVSPFGRNNQGRNIAGVEAAAQGIFGKPAKDLTVPQAAFIAG     240
pbp1b_40747      ALALERNMTKEEILTIYLNVSPFGRNNQGRNIAGVEAAAQGIFGKPAKDLTVPQAAFIAG     240
pbp1b_41126      ALALERNMTKEEILTIYLNVSPFGRNNQGRNIAGVEAAAQGIFGKPAKDLTVPQAAFIAG     240
pbp1b_30190      ALALERNMTKEEILTIYLNVSPFGRNNQGRNIAGVEAAAQGIFGKPAKDLTVPQAAFIAG     240
                 ************************************************************

pbp1b_32098      LPQSPIVYSPYASDGTRKADEDMVYGIERYQDVLFNMYRASFLTKEEYETYKAYDIKQDF     300
pbp1b_33329      LPQSPIVYSPYASDGTRKADEDMVYGIERYQDVLFNMYRASFLTKEEYETYKAYDIKQDF     300
pbp1B_P1/7       LPQSPIVYSPYASDGTRKSDEDMVYGIERYQDVLFNMYRASFLTKEEYETYKAYDIKQDF     300
pbp1b_32494      LPQSPIVYSPYASDGTRKSDEDMVYGIERYQDVLFNMYRASFLTKEEYETYKAYDIKQDF     300
pbp1b_22715      LPQSPIVYSPYASDGTRKSDEDMVYGVERYQDVLFNMYRASFLTKEEYETYKAYDIKQDF     300
pbp1b_37647      LPQSPIVYSPYASDGTRKSDEDMVYGVERYQDVLFNMYRASFLTKEEYETYKAYDIKQDF     300
pbp1b_40085      LPQSPIVYSPYASDGTRKSDEDMVYGVERYQDVLFNMYRASFLTKEEYETYKAYDIKQDF     300
pbp1b_40484      LPQSPIVYSPYASDGTRKSDEDMVYGVERYQDVLFNMYRASFLTKEEYETYKAYDIKQDF     300
pbp1b_40747      LPQSPIVYSPYASDGTRKSDEDMVYGVERYQDVLFNMYRASFLTKEEYETYKAYDIKQDF     300
pbp1b_41126      LPQSPIVYSPYASDGTRKSDEDMVYGVERYQDVLFNMYRASFLTKEEYETYKAYDIKQDF     300
pbp1b_30190      LPQSPIVYSPYASDGTRKSDEDMVYGVERYQDVLFNMYRASFLTKEEYETYKAYDIKQDF     300
                 ******************:*******:*********************************
```

```
pbp1b_32098      IAPAPVMADTKDYLYYEVMEEAQEVMFDYLVKRDKVSENDLKNDETKASYEELAKQELSQ      360
pbp1b_33329      IAPAPVMADTKDYLYYEVMEEAQEVMFDYLVKRDKVSENDLKNDETKASYEELAKQELSQ      360
pbp1B_P1/7       IAPAPVMADTKDYLYYKVMEEAQEVMFDYLVKRDKVSKNDLKNDETKASYEELAKQELSQ      360
pbp1b_32494      IAPAPVMADTKDYLYYKVMEEAQEVMFDYLVKRDKVSKNDLKNDETKASYEELAKQELSQ      360
pbp1b_22715      IAPAPVMADTKDYLYYKVMEEAQEVMFDYLVKRDKVSKNDLKNDETKASYEELAKQELSQ      360
pbp1b_37647      IAPAPVMADTKDYLYYKVMEEAQEVMFDYLVKRDKVSKNDLKNDETKASYEELAKQELSQ      360
pbp1b_40085      IAPAPVMADTKDYLYYKVMEEAQEVMFDYLVKRDKVSKNDLKNDETKASYEELAKQELSQ      360
pbp1b_40484      IAPAPVMADTKDYLYYKVMEEAQEVMFDYLVKRDKVSKNDLKNDETKASYEELAKQELSQ      360
pbp1b_40747      IAPAPVMADTKDYLYYKVMEEAQEVMFDYLVKRDKVSKNDLKNDETKASYEELAKQELSQ      360
pbp1b_41126      IAPAPVMADTKDYLYYKVMEEAQEVMFDYLVKRDKVSKNDLKNDETKASYEELAKQELSQ      360
pbp1b_30190      IAPAPVMADTKDYLYYKVMEEAQEVMFDYLVKRDKVSKNDLKNDETKASYEELAKQELSQ      360
                 ****************:********************:**********************

pbp1b_32098      GGYTIKSTVDQGIYAAMQSVVANYGSVLDDGNEYVETGSVLIDNATGAILGFVGGRDYAT      420
pbp1b_33329      GGYTIKSTVDQGIYAAMQSVVANYGSVLDDGNEYVETGSVLIDNATGAILGFVGGRDYAT      420
pbp1B_P1/7       GGYTIKSTVDQKIYAAMQSVVANYGSVLDDGNEYVETGSVLIDNATGAILGFVGGRDYAT      420
pbp1b_32494      GGYTIKSTVDQKIYAAMQSVVANYGSVLDDGNEYVETGSVLIDNATGAILGFVGGRDYAT      420
pbp1b_22715      GGYTIKSTVDQGIYAAMQSVVANYGSVLDDGNEYVETGSVLIDNATGAILGFVGGRDYST      420
pbp1b_37647      GGYTIKSTVDQGIYAAMQSVVANYGSVLDDGNEYVETGSVLIDNATGAILGFVGGRDYST      420
pbp1b_40085      GGYTIKSTVDQGIYAAMQSVVANYGSVLDDGNEYVETGSVLIDNATGAILGFVGGRDYST      420
pbp1b_40484      GGYTIKSTVDQGIYAAMQSVVANYGSVLDDGNEYVETGSVLIDNATGAILGFVGGRDYST      420
pbp1b_40747      GGYTIKSTVDQGIYAAMQSVVANYGSVLDDGNEYVETGSVLIDNATGAILGFVGGRDYST      420
pbp1b_41126      GGYTIKSTVDQGIYAAMQSVVANYGSVLDDGNEYVETGSVLIDNATGAILGFVGGRDYST      420
pbp1b_30190      GGYTIKSTVDQGIYAAMQSVVANYGSVLDDGNEYVETGSVLIDNATGAILGFVGGRDYST      420
                 *********** **********************************************:*

pbp1b_32098      NQNNHAFDTLRSPASTIKPLLAYGIAIDQGLIGSASILSDYPTNFSSGQPIMYGSGRGTG      480
pbp1b_33329      NQNNHAFDTLRSPASTIKPLLAYGIAIDQGLIGSASILSDYPTNFSSGQPIMYGSGRGTG      480
pbp1B_P1/7       NQNNHAFDTLRSPASTIKPLLAYGIAIDQGLIGSASILSDYPTNFSSGQPIMYGSGRGTG      480
pbp1b_32494      NQNNHAFDTLRSPASTIKPLLAYGIAIDQGLIGSASILSDYPTNFSSGQPIMYGSGRGTG      480
pbp1b_22715      NQNNHAFDTLRSPASTIKPLLAYGIAIDQGLIGSASILSDYPTNFSSGQPIMYGSRRGTG      480
pbp1b_37647      NQNNHAFDTLRSPASTIKPLLAYGIAIDQGLIGSASILSDYPTNFSSGQPIMYGSRRGTG      480
pbp1b_40085      NQNNHAFDTLRSPASTIKPLLAYGIAIDQGLIGSASILSDYPTNFSSGQPIMYGSRRGTG      480
pbp1b_40484      NQNNHAFDTLRSPASTIKPLLAYGIAIDQGLIGSASILSDYPTNFSSGQPIMYGSRRGTG      480
pbp1b_40747      NQNNHAFDTLRSPASTIKPLLAYGIAIDQGLIGSASILSDYPTNFSSGQPIMYGSRRGTG      480
pbp1b_41126      NQNNHAFDTLRSPASTIKPLLAYGIAIDQGLIGSASILSDYPTNFSSGQPIMYGSRRGTG      480
pbp1b_30190      NQNNHAFDTLRSPASTIKPLLAYGIAIDQGLIGSASILSDYPTNFSSGQPIMYGSGRGTG      480
                 ******************************************************* ****

pbp1b_32098      MMNLQTAIDRSVNIPAFWTYKMMRNAGVDAKAYMDKMNYHIPMYDIESVPLGGGVEISVL      540
pbp1b_33329      MMNLQTAIDRSVNIPAFWTYKMMRNAGVDAKAYMDKMNYHIPMYDIESVPLGGGVEISVL      540
pbp1B_P1/7       MMNLQTAIDRSVNIPAFWTYKMMRNAGVDAKAYMDKMNYHIPMYDIESVPLGGGVEISVL      540
pbp1b_32494      MMNLQTAIDRSVNIPAFWTYKMMRNAGVDAKAYMDKMNYHIPMYDIESVPLGGGVEISVL      540
pbp1b_22715      MMNLQTAIDRSVNIPAFWTYKMMRNAGVDAKAYMDKMNYHIPMYDIESVPLGGGVEISVL      540
pbp1b_37647      MMNLQTAIDRSVNIPAFWTYKMMRNAGVDAKAYMDKMNYHIPMYDIESVPLGGGVEISVL      540
pbp1b_40085      MMNLQTAIDRSVNIPAFWTYKMMRNAGVDAKAYMDKMNYHIPMYDIESVPLGGGVEISVL      540
pbp1b_40484      MMNLQTAIDRSVNIPAFWTYKMMRNAGVDAKAYMDKMNYHIPMYDIESVPLGGGVEISVL      540
pbp1b_40747      MMNLQTAIDRSVNIPAFWTYKMMRNAGVDAKAYMDKMNYHIPMYDIESVPLGGGVEISVL      540
pbp1b_41126      MMNLQTAIDRSVNIPAFWTYKMMRNAGVDAKAYMDKMNYHIPMYDIESVPLGGGVEISVL      540
pbp1b_30190      MMNLQTAIDRSINIPAFWTYKMMRNAGVDAKAYMDKMNYHIPMYDIESVPLGGGVEISVL      540
                 ***********:************************************************

pbp1b_32098      TNTNAYQTLANGGVYNKHYIVESITASDGTVVYQHEAAPVQVYSKATASIMNQLLRQVVN      600
pbp1b_33329      TNTNAYQTLANGGVYNKHYIVESITASDGTVVYQHEAAPVQVYSKATASIMNQLLRQVVN      600
pbp1B_P1/7       TNTNAYQTLANGGVYNKHYIVESITASDGTVVYQHEATPVQVYSKATASIMNQLLRQVVN      600
pbp1b_32494      TNTNAYQTLANGGVYNKHYIVESITASDGTVVYQHEATPVQVYSKATASIMNQLLRQVVN      600
pbp1b_22715      TNTNAYQTLANGGVYNKHYIVESITASDGTVVYQHEAAPVQVYSKATASIMNQLLRQVVN      600
pbp1b_37647      TNTNAYQTLANGGVYNKHYIVESITASDGTVVYQHEAAPVQVYSKATASIMNQLLRQVVN      600
pbp1b_40085      TNTNAYQTLANGGVYNKHYIVESITASDGTVVYQHEAAPVQVYSKATASIMNQLLRQVVN      600
pbp1b_40484      TNTNAYQTLANGGVYNKHYIVESITASDGTVVYQHEAAPVQVYSKATASIMNQLLRQVVN      600
pbp1b_40747      TNTNAYQTLANGGVYNKHYIVESITASDGTVVYQHEAAPVQVYSKATASIMNQLLRQVVN      600
pbp1b_41126      TNTNAYQTLANGGVYNKHYIVESITASDGTVVYQHEAAPVQVYSKATASIMNQLLRQVVN      600
pbp1b_30190      TNTNAYQTLANGGVYNKHYIVESITASDGTVVYQHEAAPVQVYSKATASIMNQLLRQVVN      600
                 *************************************:**********************
```

```
pbp1b_32098      SGYTTTFKSRLSGLNPQAASSDFVGKTGTSNEVNDVWLMLSTPRVTLGTWAGNDDNSEMY     660
pbp1b_33329      SGYTTTFKSRLSGLNPQAASSDFVGKTGTSNEVNDVWLMLSTPRVTLGTWAGNDDNSEMY     660
pbp1B_P1/7       SGYTTTFKSRLSGLNPQAASSDFVGKTGTSNEVNDVWLMLSTPRVTLGTWAGNDDNSEMY     660
pbp1b_32494      SGYTTTFKSRLSGLNPQAASSDFVGKTGTSNEVNDVWLMLSTPRVTLGTWAGNDDNSEMY     660
pbp1b_22715      SGYTTTFKSRLSGLNPQAASSDFVGKTGTSNEVNDVWLMLSTPRVTLGTWAGNDDNSEMY     660
pbp1b_37647      SGYTTTFKSRLSGLNPQAASSDFVGKTGTSNEVNDVWLMLSTPRVTLGTWAGNDDNSEMY     660
pbp1b_40085      SGYTTTFKSRLSGLNPQAASSDFVGKTGTSNEVNDVWLMLSTPRVTLGTWAGNDDNSEMY     660
pbp1b_40484      SGYTTTFKSRLSGLNPQAASSDFVGKTGTSNEVNDVWLMLSTPRVTLGTWAGNDDNSEMY     660
pbp1b_40747      SGYTTTFKSRLSGLNPQAASSDFVGKTGTSNEVNDVWLMLSTPRVTLGTWAGNDDNSEMY     660
pbp1b_41126      SGYTTTFKSRLSGLNPQAASSDFVGKTGTSNEVNDVWLMLSTPRVTLGTWAGNDDNSEMY     660
pbp1b_30190      SGYTTTFKSRLSGLNPQAASSDFVGKTGTSNEVNDVWLMLSTPRVTLGTWAGNDDNSEMY     660
                 ************************************************************

pbp1b_32098      VWTGYHNNSQYVAHMVDALYNVNADMFAGKFELDSSVIASSVVASTGQRAGTTQVNGRQV     720
pbp1b_33329      VWTGYHNNSQYVAHMVDALYNVNADMFAGKFELDSSVIASSVVASTGQRAGTTQVNGRQV     720
pbp1B_P1/7       VWTGYHNNSQYVAHMVDALYNVNADMFAGKFELDSSVIASSVVASTGQRAGTTQVNGRQV     720
pbp1b_32494      VWTGYHNNSQYVAHMVDALYNVNADMFAGKFELDSSVIASSVVASTGQRAGTTQVNGRQV     720
pbp1b_22715      VWTGYHNNSQYVAHMVDALYNVNADMFAGKFELDSSVIASSVVASTGQRAGTTQVNGRQV     720
pbp1b_37647      VWTGYHNNSQYVAHMVDALYNVNADMFAGKFELDSSVIASSVVASTGQRAGTTQVNGRQV     720
pbp1b_40085      VWTGYHNNSQYVAHMVDALYNVNADMFAGKFELDSSVIASSVVASTGQRAGTTQVNGRQV     720
pbp1b_40484      VWTGYHNNSQYVAHMVDALYNVNADMFAGKFELDSSVIASSVVASTGQRAGTTQVNGRQV     720
pbp1b_40747      VWTGYHNNSQYVAHMVDALYNVNADMFAGKFELDSSVIASSVVASTGQRAGTTQVNGRQV     720
pbp1b_41126      VWTGYHNNSQYVAHMVDALYNVNADMFAGKFELDSSVIASSVVASTGQRAGTTQVNGRQV     720
pbp1b_30190      VWTGYHNNSQYVAHMVDALYNVNADMFAGKFELDSSVIASSVVASTGQRAGTTQVNGRQV     720
                 ************************************************************

pbp1b_32098      TVGGAMTTSYWAKNGAPVTSYNFMVGGTDSDRAQAWNTIIGSQTPTSSSSTQRSSSTRTS     780
pbp1b_33329      TVGGAMTTSYWAKNGAPVTSYNFMVGGTDSDRAQAWNTIIGSQTPTSSSSTQRSSSTRTS     780
pbp1B_P1/7       TVGGAMTTSYWAKNGAPVTSYNFMVGGTDSDRAQAWNTIIGSQTATSSSSTQRSSSTRSS     780
pbp1b_32494      TVGGAMTTSYWAKNGAPVTSYNFMVGGTDSDRAQAWNTIIGSQTATSSSSTQRSSSTRSS     780
pbp1b_22715      TVGGAMTTSYWAKNGAPVTSYNFMVGGTDSDRAQAWNTIIGSQTPTSSSSTQRSSSTRSS     780
pbp1b_37647      TVGGAMTTSYWAKNGAPVTSYNFMVGGTDSDRAQAWNTIIGSQTPTSSSSTQRSSSTRSS     780
pbp1b_40085      TVGGAMTTSYWAKNGAPVTSYNFMVGGTDSDRAQAWNTIIGSQTPTSSSSTQRSSSTRSS     780
pbp1b_40484      TVGGAMTTSYWAKNGAPVTSYNFMVGGTDSDRAQAWNTIIGSQTPTSSSSTQRSSSTRSS     780
pbp1b_40747      TVGGAMTTSYWAKNGAPVTSYNFMVGGTDSDRAQAWNTIIGSQTPTSSSSTQRSSSTRSS     780
pbp1b_41126      TVGGAMTTSYWAKNGAPVTSYNFMVGGTDSDRAQAWNTIIGSQTPTSSSSTQRSSSTRSS     780
pbp1b_30190      TVGGAMTTSYWAKNGAPVTSYNFMVGGTDSDRAQAWNTIIGSQTPTSSSSTQRSSSTRSS     780
                 ******************************************** *************:*

pbp1b_32098      TSSSANNTNQSSETQTATSEQANNDSP     807
pbp1b_33329      TSSSANNTNQSSETQTATSEQANNDSP     807
pbp1B_P1/7       TSSSANNTNQSSETQTATSEQANNDSP     807
pbp1b_32494      TSSSANNTNQSSETQTATSEQANNDSP     807
pbp1b_22715      TSSSANNTNQSSETQTATSEQANNDSP     807
pbp1b_37647      TSSSANNTNQSSETQTATSEQANNDSP     807
pbp1b_40085      TSSSANNTNQSSETQTATSEQANNDSP     807
pbp1b_40484      TSSSANNTNQSSETQTATSEQANNDSP     807
pbp1b_40747      TSSSANNTNQSSETQTATSEQANNDSP     807
pbp1b_41126      TSSSANNTNQSSETQTATSEQANNDSP     807
pbp1b_30190      TSSSANNTNQSSETQTATSEQANNDSP     807
                 ***************************
```
