# Supplementary material for: Non-Penicillin-Susceptible Streptococcus suis Isolated from Humans
Source: Pathogens. 2021 Sep 13;10(9):1178. doi: 10.3390/pathogens10091178 (PMC8471365; doi:10.3390/pathogens10091178)
Supplement: Supplementary file 1 [file pathogens-10-01178-s001.zip › Supplementary file 3_pbp2a alignment result.pdf]

## Alignment of pbp2A sequence

```
pbp2a_32098     MDDLQHQPIEVPEDFQIDHEDRPSRRRRGNRGSVDKPKKKSRIPEPIRKFWRRYQLTKIL     60
pbp2a_33329     MDDLQHQPIEVPEDFQIDHEDRPSRRRRGNRGSVDKPKKKSRIPEPIRKFWRRYQLTKIL     60
pbp2a_22715     MDDLQHQPIEVPEDFQIDNEDQPSRRRRGNRGSVDKPKKKSRIPEPIRKFWRRYQLTKIL     60
pbp2a_37647     MDDLQHQPIEVPEDFQIDNEDQPSRRRRGNRGSVDKPKKKSRIPEPIRKFWRRYQLTKIL     60
pbp2a_40085     MDDLQHQPIEVPEDFQIDNEDQPSRRRRGNRGSVDKPKKKSRIPEPIRKFWRRYQLTKIL     60
pbp2a_40484     MDDLQHQPIEVPEDFQIDNEDQPSRRRRGNRGSVDKPKKKSRIPEPIRKFWRRYQLTKIL     60
pbp2a_40747     MDDLQHQPIEVPEDFQIDNEDQPSRRRRGNRGSVDKPKKKSRIPEPIRKFWRRYQLTKIL     60
pbp2a_41126     MDDLQHQPIEVPEDFQIDNEDQPSRRRRGNRGSVDKPKKKSRIPEPIRKFWRRYQLTKIL     60
pbp2A_P1/7      MDDLQHQPIEVPEDFQIDHEDRPSRRHRGNRGSIDKPKKKSRIPEPIRKFWRRYQLTKIL     60
pbp2a_32494     MDDLQHQPIEVPEDFQIDHEDRPSRRHRGNRGSIDKPKKKSRIPEPIRKFWRRYQLTKIL     60
pbp2a_30190     MDDLQHQPIEVPEDFQIDHEDRPSRRRRGNRGSIDKPKKKSRIPEPIRKFWRRYQLTKIL     60
                ******************:**:****:******:**************************

pbp2a_32098     IILMVLTVLTVGGYLFFLAKTANVGDLQQALKATTIIYDKDGAEAGTLSGQKGTYVELDA     120
pbp2a_33329     IILMVLTVLTVGGYLFFLAKTANVGDLQQALKATTIIYDKDGAEAGTLSGQKGTYVELDA     120
pbp2a_22715     IILMVLTVLTVGGYLFFLAKTANVGDLQQALKATTIIYDKDGAEAGTLSGQKGTYVELDA     120
pbp2a_37647     IILMVLTVLTVGGYLFFLAKTANVGDLQQALKATTIIYDKDGAEAGTLSGQKGTYVELDA     120
pbp2a_40085     IILMVLTVLTVGGYLFFLAKTANVGDLQQALKATTIIYDKDGAEAGTLSGQKGTYVELDA     120
pbp2a_40484     IILMVLTVLTVGGYLFFLAKTANVGDLQQALKATTIIYDKDGAEAGTLSGQKGTYVELDA     120
pbp2a_40747     IILMVLTVLTVGGYLFFLAKTANVGDLQQALKATTIIYDKDGAEAGTLSGQKGTYVELDA     120
pbp2a_41126     IILMVLTVLTVGGYLFFLAKTANVGDLQQALKATTIIYDKDGAEAGTLSGQKGTYVELDA     120
pbp2A_P1/7      IILMVLTVLTVGGYLFFLAKTANVGDLQQALKATTIIYDKDGAEAGTLSGQKGTYVELDA     120
pbp2a_32494     IILMVLTVLTVGGYLFFLAKTANVGDLQQALKATTIIYDKDGAEAGTLSGQKGTYVELDA     120
pbp2a_30190     IILMVLTVLTVGGYLFFLAKTANVGDLQQALKATTIIYDKDGAEAGTLSGQKGTYVELDA     120
                ************************************************************

pbp2a_32098     VSDNLENAVIATEDRSFYKNSGINYQRTILAALTLGRSGGGSTITQQLAKNAFLTQDQTI     180
pbp2a_33329     VSDNLENAVIATEDRSFYKNSGINYQRTILAALTLGRSGGGSTITQQLAKNAFLTQDQTI     180
pbp2a_22715     VSDNLENAVIATEDRSFYKNSGINYQRTILAALTLGRSGGGSTITQQLAKNAFLTQDQTI     180
pbp2a_37647     VSDNLENAVIATEDRSFYKNSGINYQRTILAALTLGRSGGGSTITQQLAKNAFLTQDQTI     180
pbp2a_40085     VSDNLENAVIATEDRSFYKNSGINYQRTILAALTLGRSGGGSTITQQLAKNAFLTQDQTI     180
pbp2a_40484     VSDNLENAVIATEDRSFYKNSGINYQRTILAALTLGRSGGGSTITQQLAKNAFLTQDQTI     180
pbp2a_40747     VSDNLENAVIATEDRSFYKNSGINYQRTILAALTLGRSGGGSTITQQLAKNAFLTQDQTI     180
pbp2a_41126     VSDNLENAVIATEDRSFYKNSGINYQRTILAALTLGRSGGGSTITQQLAKNAFLTQDQTI     180
pbp2A_P1/7      ISDNLENAVIATEDRSFYKNSGINYQRTILAALTLGRSGGGSTITQQLAKNAFLTQDQTI     180
pbp2a_32494     ISDNLENAVIATEDRSFYKNSGINYQRTILAALTLGRSGGGSTITQQLAKNAFLTQDQTI     180
pbp2a_30190     VSDNLENAVIATEDRSFYKNSGINYQRTILAALTLGRSGGGSTITQQLAKNAFLTQDQTI     180
                :***********************************************************

pbp2a_32098     SRKAREFFLALEINKKYSKQEILTMYLNNAYFGNGVWGVEDASQKYFGIPASDLTLEQAA     240
pbp2a_33329     SRKAREFFLALEINKKYSKQEILTMYLNNAYFGNGVWGVEDASQKYFGIPASDLTLEQAA     240
pbp2a_22715     SRKAREFFLALEINKKYSKQEILTMYLNNAYFGNGVWGVEDASQKYFGIPASDLTLEQAA     240
pbp2a_37647     SRKAREFFLALEINKKYSKQEILTMYLNNAYFGNGVWGVEDASQKYFGIPASDLTLEQAA     240
pbp2a_40085     SRKAREFFLALEINKKYSKQEILTMYLNNAYFGNGVWGVEDASQKYFGIPASDLTLEQAA     240
pbp2a_40484     SRKAREFFLALEINKKYSKQEILTMYLNNAYFGNGVWGVEDASQKYFGIPASDLTLEQAA     240
pbp2a_40747     SRKAREFFLALEINKKYSKQEILTMYLNNAYFGNGVWGVEDASQKYFGIPASDLTLEQAA     240
pbp2a_41126     SRKAREFFLALEINKKYSKQEILTMYLNNAYFGNGVWGVEDASQKYFGIPASDLTLEQAA     240
pbp2A_P1/7      SRKAREFFLALEINKKYSKQEILTMYLNNAYFGNGVWGVEDASQKYFGIPASNLTLEQAA     240
pbp2a_32494     SRKAREFFLALEINKKYSKQEILTMYLNNAYFGNGVWGVEDASQKYFGIPASNLTLEQAA     240
pbp2a_30190     SRKAREFFLALEINKKYSKQEILTMYLNNAYFGNGVWGVEDASQKYFGIPASDLTLEQAA     240
                ****************************************************:*******

pbp2a_32098     VIAGMLKGPEIYNPYYSLENAVNRRDTVLQNMVNAGYIDQATADAGFQVDLASQLSDTYT     300
pbp2a_33329     VIAGMLKGPEIYNPYYSLENAVNRRDTVLQNMVNAGYIDQATADAGFQVDLASQLSDTYT     300
pbp2a_22715     VIAGMLKGPEIYNPYYSLENAVNRRDTVLQNMVNAGYIDQATADAGFQVDLASQLSDTYT     300
pbp2a_37647     VIAGMLKGPEIYNPYYSLENAVNRRDTVLQNMVNAGYIDQATADAGFQVDLASQLSDTYT     300
pbp2a_40085     VIAGMLKGPEIYNPYYSLENAVNRRDTVLQNMVNAGYIDQATADAGFQVDLASQLSDTYT     300
pbp2a_40484     VIAGMLKGPEIYNPYYSLENAVNRRDTVLQNMVNAGYIDQATADAGFQVDLASQLSDTYT     300
pbp2a_40747     VIAGMLKGPEIYNPYYSLENAVNRRDTVLQNMVNAGYIDQATADAGFQVDLASQLSDTYT     300
pbp2a_41126     VIAGMLKGPEIYNPYYSLENAVNRRDTVLQNMVNAGYIDQATADAGFQVDLASQLSDTYT     300
pbp2A_P1/7      VIAGMLKGPEIYNPYYSLENAVNRRDTVLQNMVNAGYIDQATADAGFQVDLASQLSDTYT     300
pbp2a_32494     VIAGMLKGPEIYNPYYSLENAVNRRDTVLQNMVNAGYIDQATADAGFQVDLASQLSDTYT     300
pbp2a_30190     VIAGMLKGPEIYNPYYSLENAVNRRDTVLQNMVNTGYIDQATADAGFQVDLASQLSDTYT     300
                **********************************:*************************
```

```
pbp2a_32098      GKQSNYSYPSYFDAVIAEAIERYGLKESDIINNGYRIYTEMDQNSQASMQVIFNDETMFP      360
pbp2a_33329      GKQSNYSYPSYFDAVIAEAIERYGLKESDIINNGYRIYTEMDQNSQASMQVIFNDETMFP      360
pbp2a_22715      GKQSNYSYPSYFDAVIAEAIERYGLKESDIINNGYRIYTEMDQNSQASMQVIFNDETMFP      360
pbp2a_37647      GKQSNYSYPSYFDAVIAEAIERYGLKESDIINNGYRIYTEMDQNSQASMQVIFNDETMFP      360
pbp2a_40085      GKQSNYSYPSYFDAVIAEAIERYGLKESDIINNGYRIYTEMDQNSQASMQVIFNDETMFP      360
pbp2a_40484      GKQSNYSYPSYFDAVIAEAIERYGLKESDIINNGYRIYTEMDQNSQASMQVIFNDETMFP      360
pbp2a_40747      GKQSNYSYPSYFDAVIAEAIERYGLKESDIINNGYRIYTEMDQNSQASMQVIFNDETMFP      360
pbp2a_41126      GKQSNYSYPSYFDAVIAEAIERYGLKESDIINNGYRIYTEMDQNSQASMQVIFNDETMFP      360
pbp2A_P1/7       GKQSNYSYPSYFDAVIAEAIERYGLKESDIINNGYRIYTEMDQNSQASMQVIFNDETMFP      360
pbp2a_32494      GKQSNYSYPSYFDAVIAEAIERYGLKESDIINNGYRIYTEMDQNSQASMQVIFNDETMFP      360
pbp2a_30190      GKQSNYSYPSYFDAVIAEAIERYGLKESDIINNGYRIYTEMDQNSQASMQVIFNDETMFP      360
                 ************************************************************

pbp2a_32098      TSSFDGTHAQAASVALDPTTGGVRALVGRVNSSEDAVFRTFNFATQAKRSPGSTIKPLVA      420
pbp2a_33329      TSSFDGTHAQAASVALDPTTGGVRALVGRVNSSEDAVFRTFNFATQAKRSPGSTIKPLVA      420
pbp2a_22715      TSSFDGTHAQAASVALDPRTGGVRALVGRINSSEDAVFRTFNFATQAKRSPGSTIKPLVA      420
pbp2a_37647      TSSFDGTHAQAASVALDPRTGGVRALVGRINSSEDAVFRTFNFATQAKRSPGSTIKPLVA      420
pbp2a_40085      TSSFDGTHAQAASVALDPRTGGVRALVGRINSSEDAVFRTFNFATQAKRSPGSTIKPLVA      420
pbp2a_40484      TSSFDGTHAQAASVALDPRTGGVRALVGRINSSEDAVFRTFNFATQAKRSPGSTIKPLVA      420
pbp2a_40747      TSSFDGTHAQAASVALDPRTGGVRALVGRINSSEDAVFRTFNFATQAKRSPGSTIKPLVA      420
pbp2a_41126      TSSFDGTHAQAASVALDPRTGGVRALVGRINSSEDAVFRTFNFATQAKRSPGSTIKPLVA      420
pbp2A_P1/7       TSSFDGTHAQAASVALDPRTGGVRALVGRVNSSEDAVFRTFNFATQAKRSPGSTIKPLVA      420
pbp2a_32494      TSSFDGTHAQAASVALDPRTGGVRALVGRVNSSEDAVFRTFNFATQAKRSPGSTIKPLVA      420
pbp2a_30190      TSSFDGTHAQAASVALDPRTGGVRALVGRVNSSEDAVFRTFNFATQAKRSPGSTIKPLVA      420
                 ****************** **********:******************************

pbp2a_32098      YAPAVAAGWAIDTPLDNHTETYGDYTLHNYDYSTSDTIPMYQALALSYNLPVAYIVNTFG      480
pbp2a_33329      YAPAVAAGWAIDTPLDNHTETYGDYTLHNYDYSTSDTIPMYQALALSYNLPVAYIVNTFG      480
pbp2a_22715      YAPAVAAGWSIDMSLDNHTETYGDYTLHNYDYSTSDTIPMYQALALSYNLPVAYIVNTLG      480
pbp2a_37647      YAPAVAAGWSIDMSLDNHTETYGDYTLHNYDYSTSDTIPMYQALALSYNLPVAYIVNTLG      480
pbp2a_40085      YAPAVAAGWSIDMSLDNHTETYGDYTLHNYDYSTSDTIPMYQALALSYNLPVAYIVNTLG      480
pbp2a_40484      YAPAVAAGWSIDMSLDNHTETYGDYTLHNYDYSTSDTIPMYQALALSYNLPVAYIVNTLG      480
pbp2a_40747      YAPAVAAGWSIDMSLDNHTETYGDYTLHNYDYSTSDTIPMYQALALSYNLPVAYIVNTLG      480
pbp2a_41126      YAPAVAAGWSIDMSLDNHTETYGDYTLHNYDYSTSDTIPMYQALALSYNLPVAYIVNTLG      480
pbp2A_P1/7       YAPAVAAGWSIDMALDNHTETYGDYTLHNYDYSTSDTIPMYQALALSYNLPVAYIVNTLG      480
pbp2a_32494      YAPAVAAGWSIDMALDNHTETYGDYTLHNYDYSTSDTIPMYQALALSYNLPVAYIVNTLG      480
pbp2a_30190      YAPAVAAGWSIDMALDNHTETYGDYTLHNYDYSTSDTIPMYQALALSYNLPVAYIVNTLG      480
                 *********:**  ********************************************:*

pbp2a_32098      IDKAFEFGQKFGLDMENVEKVLGASIGSGVETNPLQMAQAYSAFANDGVMKDAHLITRIE      540
pbp2a_33329      IDKAFEFGQKFGLDMENVEKVLGASIGSGVETNPLQMAQAYSAFANDGVMKDAHLITRIE      540
pbp2a_22715      IDKAFEYGQKFGLDMENVEKVLGVSIGSGVETNPLQMAQAYATFANKGVMKDAHLITRIE      540
pbp2a_37647      IDKAFEYGQKFGLDMENVEKVLGVSIGSGVETNPLQMAQAYATFANKGVMKDAHLITRIE      540
pbp2a_40085      IDKAFEYGQKFGLDMENVEKVLGVSIGSGVETNPLQMAQAYATFANKGVMKDAHLITRIE      540
pbp2a_40484      IDKAFEYGQKFGLDMENVEKVLGVSIGSGVETNPLQMAQAYATFANKGVMKDAHLITRIE      540
pbp2a_40747      IDKAFEYGQKFGLDMENVEKVLGVSIGSGVETNPLQMAQAYATFANKGVMKDAHLITRIE      540
pbp2a_41126      IDKAFEYGQKFGLDMENVEKVLGVSIGSGVETNPLQMAQAYATFANKGVMKDAHLITRIE      540
pbp2A_P1/7       IDKAFEYGQKFGLDMENVEKVLGVSIGSGVETNPLQMAQAYATFANKGVMKDAHLITRIE      540
pbp2a_32494      IDKAFEYGQKFGLDMENVEKVLGVSIGSGVETNPLQMAQAYATFANKGVMKDAHLITRIE      540
pbp2a_30190      IDKAFEYGQKFGLDMENVEKVLGVSIGSGVETNPLQMAQAYATFANKGVMKDAHLITRIE      540
                 ******:****************.*****************::***.*************

pbp2a_32098      TASGKVLAQHRETSTKVIDRSVADKMTSMMLGTYTNGLGVSADATNYYLAGKTGTTETSF      600
pbp2a_33329      TASGKVLAQHRETSTKVIDRSVADKMTSMMLGTYTNGLGVSADATNYYLAGKTGTTETSF      600
pbp2a_22715      TASGKVLAEHRETSTKVIDRSVADKMTAMMLGTYTNGTGVTADTANYHIAGKTGTTETSF      600
pbp2a_37647      TASGKVLAEHRETSTKVIDRSVADKMTAMMLGTYTNGTGVTADTANYHIAGKTGTTETSF      600
pbp2a_40085      TASGKVLAEHRETSTKVIDRSVADKMTAMMLGTYTNGTGVTADTANYHIAGKTGTTETSF      600
pbp2a_40484      TASGKVLAEHRETSTKVIDRSVADKMTAMMLGTYTNGTGVTADTANYHIAGKTGTTETSF      600
pbp2a_40747      TASGKVLAEHRETSTKVIDRSVADKMTAMMLGTYTNGTGVTADTANYHIAGKTGTTETSF      600
pbp2a_41126      TASGKVLAEHRETSTKVIDRSVADKMTAMMLGTYTNGTGVTADTANYHIAGKTGTTETSF      600
pbp2A_P1/7       TASGKVLAEHRETSTKVIDRAVADKMTAMMLGTYTNGTGVTADTANYHIAGKTGTTETSF      600
pbp2a_32494      TASGKVLAEHRETSTKVIDRAVADKMTAMMLGTYTNGTGVTADTANYHIAGKTGTTETSF      600
pbp2a_30190      TASGKVLAEHRETSTKVIDRSVADKMTAMMLGTYTNGTGVTADTANYHIAGKTGTTETSF      600
                 ********:***********:******:*********  **:**::**::***********
```

```
pbp2a_32098      DVNLVNDQWYIAYTPDLVISQWVGFEQTDENHYIDSSGYWMAPTVFRTVADAILPNTAGT    660
pbp2a_33329      DVNLVNDQWYIAYTPDLVISQWVGFEQTDENHYIDSSGYWMAPTVFRTVADAILPNTAGT    660
pbp2a_22715      DVNLVNDQWHIAYTPDLVISQWVGFEQTDENHYIDSNSYWKAPSVFQTVANSILPYTAGT    660
pbp2a_37647      DVNLVNDQWHIAYTPDLVISQWVGFEQTDENHYIDSNSYWKAPSVFQTVANSILPYTAGT    660
pbp2a_40085      DVNLVNDQWHIAYTPDLVISQWVGFEQTDENHYIDSNSYWKAPSVFQTVANSILPYTAGT    660
pbp2a_40484      DVNLVNDQWHIAYTPDLVISQWVGFEQTDENHYIDSNSYWKAPSVFQTVANSILPYTAGT    660
pbp2a_40747      DVNLVNDQWHIAYTPDLVISQWVGFEQTDENHYIDSNSYWKAPSVFQTVANSILPYTAGT    660
pbp2a_41126      DVNLVNDQWHIAYTPDLVISQWVGFEQTDENHYIDSNSYWKAPSVFQTVANSILPYTAGT    660
pbp2A_P1/7       DVNLVNDQWHIAYTPDLVISQWVGFEQTDENHYIDSSSYWKAPSVFQTVANSILPYTAGS    660
pbp2a_32494      DVNLVNDQWHIAYTPDLVISQWVGFEQTDENHYIDSSSYWKAPSVFQTVANSILPYTAGS    660
pbp2a_30190      DVNLVNDQWHIAYTPDLVISQWVGFEQTDENHYIDSSSYWKAPSVFQTVANSILPYTAGT    660
                 *********:**************************..** **:**:***::*** ***:

pbp2a_32098      SFDVENAYVQNGIVEVAPDEAAQTESSLGQEQVQEISDQAKNLIEQTKQNLIDAQLPERA    720
pbp2a_33329      SFDVENAYVQNGIVEVAPDEAAQTESSLGQEQVQEISDQAKNLIEQTKQNLIDAQLPERA    720
pbp2a_22715      KFEVENAYAQNGIVEVAPDEAAQTESSLGQEQVQEISEQAKNLIEQTKQNLIDAQLPERA    720
pbp2a_37647      KFEVENAYAQNGIVEVAPDEAAQTESSLGQEQVQEISEQAKNLIEQTKQNLIDAQLPERA    720
pbp2a_40085      KFEVENAYAQNGIVEVAPDEAAQTESSLGQEQVQEISEQAKNLIEQTKQNLIDAQLPERA    720
pbp2a_40484      KFEVENAYAQNGIVEVAPDEAAQTESSLGQEQVQEISEQAKNLIEQTKQNLIDAQLPERA    720
pbp2a_40747      KFEVENAYAQNGIVEVAPDEAAQTESSLGQEQVQEISEQAKNLIEQTKQNLIDAQLPERA    720
pbp2a_41126      KFEVENAYAQNGIVEVAPDEAAQTESSLGQEQVQEISEQAKNLIEQTKQNLIDAQLPERA    720
pbp2A_P1/7       KFEVENAYAQNGIVEVAPDEAAQTESSLGQEQVQEISEQAKNLIEQTKQNLIDAQLPERA    720
pbp2a_32494      KFEVENAYAQNGIVEVAPDEAAQTESSLGQEQVQEISEQAKNLIEQTKQNLIDAQLPERA    720
pbp2a_30190      KFEVENAYAQNGIVEVAPDEAAQTESSLGQEQVQEISEQAKNLIEQTKQNLIDAQLPERA    720
                 .*:*****.****************************:**********************

pbp2a_32098      KTLWDNITSWFGELSNP        737
pbp2a_33329      KTLWDNITSWFGELSNP        737
pbp2a_22715      KTLWDNITSWFGELSNP        737
pbp2a_37647      KTLWDNITSWFGELSNP        737
pbp2a_40085      KTLWDNITSWFGELSNP        737
pbp2a_40484      KTLWDNITSWFGELSNP        737
pbp2a_40747      KTLWDNITSWFGELSNP        737
pbp2a_41126      KTLWDNITSWFGELSNP        737
pbp2A_P1/7       KTLWDNITSWFGELSNP        737
pbp2a_32494      KTLWDNITSWFGELSNP        737
pbp2a_30190      KTLWDNITSWFGELSNP        737
                 *****************
```
