# Supplementary material for: Non-Penicillin-Susceptible Streptococcus suis Isolated from Humans
Source: Pathogens. 2021 Sep 13;10(9):1178. doi: 10.3390/pathogens10091178 (PMC8471365; doi:10.3390/pathogens10091178)
Supplement: Supplementary file 1 [file pathogens-10-01178-s001.zip › Supplementary file 4_pbp2b alignment result.pdf]

Alignment of pbp2B sequence

```
pbp2b_32098      MKKRKNKPLEHDGNFISLRLNILFSIVIFLFLVLILRLADMQIINHDFYSNKLSTASQKI    60
pbp2b_33329      MKKRKNKPLEHDGNFISLRLNILFSIVIFLFLVLILRLADMQIINHDFYSNKLSTASQKI    60
pbp2b_37647      MKKRKNKPLEHDGNFISLRLNILFSIVIFLFLVLILRLADMQIINHDFYSNKLSTASQKI    60
pbp2b_40085      MKKRKNKPLEHDGNFISLRLNILFSIVIFLFLVLILRLADMQIINHDFYSNKLSTASQKI    60
pbp2b_40484      MKKRKNKPLEHDGNFISLRLNILFSIVIFLFLVLILRLADMQIINHDFYSNKLSTASQKI    60
pbp2b_40747      MKKRKNKPLEHDGNFISLRLNILFSIVIFLFLVLILRLADMQIINHDFYSNKLSTASQKI    60
pbp2b_41126      MKKRKNKPLEHDGNFISLRLNILFSIVIFLFLVLILRLADMQIINHDFYSNKLSTASQKI    60
pbp2b_30190      MKKRKNKPLEHDGNFISLRLNILFSIVIFLFLVLILRLADMQIINHDFYSNKLSTASQKI    60
pbp2b_P1/7       MKKRKNKPLEHDGNFISLRLNILFSIVIFLFLVLILRLADMQIINHDFYSNKLSTASQKI    60
pbp2b_32494      MKKRKNKPLEHDGNFISLRLNILFSIVIFLFLVLILRLADMQIINHDFYSNKLSTASQKI    60
pbp2b_22715      MKKRKNKPLEHDGNFISLRLNILFSIVIFLFLVLILRLADMQIINHDFYSNKLSTASQKI    60
                 ************************************************************

pbp2b_32098      ISNGSIRGQIYDAQGKPLVENEIQQVVSFTRSNKMSAQEMKEVANKLLQWVNVSDVTITQ    120
pbp2b_33329      ISNGSIRGQIYDAQGKPLVENEIQQVVSFTRSNKMSAQEMKEVANKLLQWVNVSDVTITQ    120
pbp2b_37647      ISNGSIRGQIYDAQGKPLVENEIQQVVSFTRSNKMSAQEMKEVANKLLQWVNVSDVTITQ    120
pbp2b_40085      ISNGSIRGQIYDAQGKPLVENEIQQVVSFTRSNKMSAQEMKEVANKLLQWVNVSDVTITQ    120
pbp2b_40484      ISNGSIRGQIYDAQGKPLVENEIQQVVSFTRSNKMSAQEMKEVANKLLQWVNVSDVTITQ    120
pbp2b_40747      ISNGSIRGQIYDAQGKPLVENEIQQVVSFTRSNKMSAQEMKEVANKLLQWVNVSDVTITQ    120
pbp2b_41126      ISNGSIRGQIYDAQGKPLVENEIQQVVSFTRSNKMSAQEMKEVANKLLQWVNVSDVTITQ    120
pbp2b_30190      ISNGSIRGQIYDAQGKPLVENEIQQVVSFTRPNKMSAQEMKEVANKLLQWVNVSDVTITQ    120
pbp2b_P1/7       ISNGSIRGQIYDAQGKPLVENEIQQVVSFTRPNKMSAQEMKEVANKLLQWVNVSDVTITQ    120
pbp2b_32494      ISNGSIRGQIYDAQGKPLVENEIQQVVSFTRPNKMSAQEMKEVANKLLQWVNVSDVTITQ    120
pbp2b_22715      ISNGSIRGQIYDAQGKPLVENEIQQVVSFTRPNKMSAQEMKEVANKLLQWVNVSDVTITQ    120
                 ******************************* ****************************

pbp2b_32098      RDKADYYLADEEVYRQVVEALPEDKKYDSDGNYLSESTIYSNAVDSIPAEALQYSEQDSK    180
pbp2b_33329      RDKADYYLADEEVYRQVVEALPEDKKYDSDGNYLSESTIYSNAVDSIPAEALQYSEQDSK    180
pbp2b_37647      RDKADYYLADEEVYRQVVEALPEDKKYDSDGNYLSESTIYSNAVDSIPAEALQYSEQDSK    180
pbp2b_40085      RDKADYYLADEEVYRQVVEALPEDKKYDSDGNYLSESTIYSNAVDSIPAEALQYSEQDSK    180
pbp2b_40484      RDKADYYLADEEVYRQVVEALPEDKKYDSDGNYLSESTIYSNAVDSIPAEALQYSEQDSK    180
pbp2b_40747      RDKADYYLADEEVYRQVVEALPEDKKYDSDGNYLSESTIYSNAVDSIPAEALQYSEQDSK    180
pbp2b_41126      RDKADYYLADEEVYRQVVEALPEDKKYDSDGNYLSESTIYSNAVDSIPAEALQYSEQDSK    180
pbp2b_30190      RDKADYYLADEEVYRQVVEALPKDKKYDSDGNYLSESTIYSNAVDSIPAEALQYSEQDNK    180
pbp2b_P1/7       RDKADYYLADEEVYRQVVEALPKDKKYDSDGNYLSESTIYSNAVDSIPAEALQYSEQDNK    180
pbp2b_32494      RDKADYYLADEEVYRQVVEALPKDKKYDSDGNYLSESTIYSNAVDSIPAEALQYSEQDNK    180
pbp2b_22715      RDKADYYLADEEVYRQVVEALPEDKKYDSDGNYLSESTIYSNAVDSIPAEALQYSEQDNK    180
                 **********************:***********************************.*

pbp2b_32098      AIELFKQMNGATYFSTVNLVTDTLTAEQVAHIVANESQLPGISTTNNWQRNILPTSLSSV    240
pbp2b_33329      AIELFKQMNGATYFSTVNLVTDTLTAEQVAHIVANESQLPGISTTNNWQRNILPTSLSSV    240
pbp2b_37647      AIELFKQMNGATYFSTVNLVTDTLTAEQVAHIVANESQLPGISTTNNWQRTILPTSLSSI    240
pbp2b_40085      AIELFKQMNGATYFSTVNLVTDTLTAEQVAHIVANESQLPGISTTNNWQRTILPTSLSSI    240
pbp2b_40484      AIELFKQMNGATYFSTVNLVTDTLTAEQVAHIVANESQLPGISTTNNWQRTILPTSLSSI    240
pbp2b_40747      AIELFKQMNGATYFSTVNLVTDTLTAEQVAHIVANESQLPGISTTNNWQRTILPTSLSSI    240
pbp2b_41126      AIELFKQMNGATYFSTVNLVTDTLTAEQVAHIVANESQLPGISTTNNWQRTILPTSLSSI    240
pbp2b_30190      AIELFKQMNGATYFSTVNLVTDTLTSEQVAHIVANESQLPGISTTNNWQRTILPTSLSSI    240
pbp2b_P1/7       AIELFKQMNGATYFSTVNLVTDTLTSEQVAHIVANESQLPGISTTNNWQRTILPTSLSSI    240
pbp2b_32494      AIELFKQMNGATYFSTVNLVTDTLTSEQVAHIVANESQLPGISTTNNWQRTILPTSLSSI    240
pbp2b_22715      AIELFKQMNGATYFSTVNLVTDTLTSEQVAHIVANESQLPGISTTNNWQRTILPTSLSSI    240
                 *************************:************************.********:

pbp2b_32098      IGTVTSEQAGLPAEDAEYYLSKGYSPNDRVGTAYLEKQYEEVLQGQREKKEINLDRNGNV    300
pbp2b_33329      IGTVTSEQAGLPAEDAEYYLSKGYSPNDRVGTAYLEKQYEEVLQGQREKKEINLDRNGNV    300
pbp2b_37647      IGTVTSEQAGLPAEDAEYYLSKGYSSNDRVGTAYLEKQYEEVLQGQREKKQINLDRNGNV    300
pbp2b_40085      IGTVTSEQAGLPAEDAEYYLSKGYSSNDRVGTAYLEKQYEEVLQGQREKKQINLDRNGNV    300
pbp2b_40484      IGTVTSEQAGLPAEDAEYYLSKGYSSNDRVGTAYLEKQYEEVLQGQREKKQINLDRNGNV    300
pbp2b_40747      IGTVTSEQAGLPAEDAEYYLSKGYSSNDRVGTAYLEKQYEEVLQGQREKKQINLDRNGNV    300
pbp2b_41126      IGTVTSEQAGLPAEDAEYYLSKGYSSNDRVGTAYLEKQYEEVLQGQREKKQINLDRNGNV    300
pbp2b_30190      IGTVTSEQAGLPAEDAEYYLSKGYSSNDRVGTAYLEKQYEEVLQGQREKKQINLDRNGNI    300
pbp2b_P1/7       IGTVTSEQAGLPAEDAEYYLSKGYSSNDRVGTAYLEKQYEEVLQGQREKKQINLDRNGNI    300
pbp2b_32494      IGTVTSEQAGLPAEDAEYYLSKGYSSNDRVGTAYLEKQYEEVLQGQREKKQINLDRNGNI    300
pbp2b_22715      IGTVTSEQAGLPAEDAEYYLSKGYSSNDRVGTAYLEKQYEEVLQGQREKKQINLDRNGNI    300
                 ************************* ************************:********:
```

```
pbp2b_32098      ESIKTIQEGQQGNNIKLTIDLAFQDGVNAILKKYFESELSTGSALYSEGVYAVALNPSTG      360
pbp2b_33329      ESIKTIQEGQQGNNIKLTIDLAFQDGVNAILKKYFESELSTGSALYSEGVYAVALNPSTG      360
pbp2b_37647      ESIETIQEGQQGNNIKLTIDLAFQDGVNAILKKYFESELATGSALYSEGVYAVALEPSTG      360
pbp2b_40085      ESIETIQEGQQGNNIKLTIDLAFQDGVNAILKKYFESELATGSALYSEGVYAVALEPSTG      360
pbp2b_40484      ESIETIQEGQQGNNIKLTIDLAFQDGVNAILKKYFESELATGSALYSEGVYAVALEPSTG      360
pbp2b_40747      ESIETIQEGQQGNNIKLTIDLAFQDGVNAILKKYFESELATGSALYSEGVYAVALEPSTG      360
pbp2b_41126      ESIETIQEGQQGNNIKLTIDLAFQDGVNAILKKYFESELATGSALYSEGVYAVALEPSTG      360
pbp2b_30190      ESIETIQEGQQGNNIKLTIDLAFQDGVNAILRKYFESELATGSALYSEGVYAVALNPSTG      360
pbp2b_P1/7       ESIETIQEGQQGNNIKLTIDLAFQDGVNAILRKYFESELATGSALYSEGVYAVALNPSTG      360
pbp2b_32494      ESIETIQEGQQGNNIKLTIDLAFQDGVNAILRKYFESELATGSALYSEGVYAVALNPSTG      360
pbp2b_22715      ESIETIQEGQQGNNIKLTIDLAFQDGVNAILRKYFESELATGSALYSEGVYAVALNPSTG      360
                 ***:***************************:*******:***************:****

pbp2b_32098      AVLAMSGYSHEKGSSTITEDALGTITSVFTPGSIVKGATISSGWENGIISGNQVLLDEPI      420
pbp2b_33329      AVLAMSGYSHEKGSSTITEDALGTITSVFTPGSIVKGATISSGWENGIISGNQVLLDEPI      420
pbp2b_37647      AVLAMSGYSHEKGAGSITEDALGTITSVFTPGSIVKGATISSGWENGVISGNQVQLDEPI      420
pbp2b_40085      AVLAMSGYSHEKGAGSITEDALGTITSVFTPGSIVKGATISSGWENGVISGNQVQLDEPI      420
pbp2b_40484      AVLAMSGYSHEKGAGSITEDALGTITSVFTPGSIVKGATISSGWENGVISGNQVQLDEPI      420
pbp2b_40747      AVLAMSGYSHEKGAGSITEDALGTITSVFTPGSIVKGATISSGWENGVISGNQVQLDEPI      420
pbp2b_41126      AVLAMSGYSHEKGAGSITEDALGTITSVFTPGSIVKGATISSGWENGVISGNQVQLDEPI      420
pbp2b_30190      AVLAMSGYSHEKGSSTITEDALGTITSVFTPGSIVKGATISSGWENGVISGNQVQLDEPI      420
pbp2b_P1/7       AVLAMSGYSHEKGSSTITEDALGTITSVFTPGSIVKGATISSGWENGVISGNQIQLDEPI      420
pbp2b_32494      AVLAMSGYSHEKGSSTITEDALGTITSVFTPGSIVKGATISSGWENGVISGNQIQLDEPI      420
pbp2b_22715      AVLAMSGYSHEKGSSTITEDALGTITSVFTPGSIVKGATISSGWENGVISGNQVQLDEPI      420
                 *************:.:*******************************:*****: *****

pbp2b_32098      YFAGSAPITSWW--AYGSFNIDATEALEYSSNVYMVKIALGLLGQTYSANMYLNDGDTLT      478
pbp2b_33329      YFAGSAPITSWW--AYGSFNIDATEALEYSSNVYMVKIALGLLGQTYSANMYLNDGDTLT      478
pbp2b_37647      YFAGSAPITSWW--AYGSFNIDATEALEYSSNAYMVKIALGLLGQTYSANMYLNDGDTLT      478
pbp2b_40085      YFAGSAPITSWW--AYGSFNIDATEALEYSSNAYMVKIALGLLGQTYSANMYLNDGDTLT      478
pbp2b_40484      YFAGSAPITSWW--AYGSFNIDATEALEYSSNAYMVKIALGLLGQTYSANMYLNDGDTLT      478
pbp2b_40747      YFAGSAPITSWW--AYGSFNIDATEALEYSSNAYMVKIALGLLGQTYSANMYLNDGDTLT      478
pbp2b_41126      YFAGSAPITSWW--AYGSFNIDATEALEYSSNAYMVKIALGLLGQTYSANMYLNDGDTLT      478
pbp2b_30190      YFAGSAPITSWYGGAYGSFNIDATEALEYSSNIYMVKIALGLLGQTYSANMYLNDGDTLK      480
pbp2b_P1/7       YFAGSAPITSWYG-AYGSFNIDATEALEYSSNIYMVKIALGLLGQTYSANMYLNDGDTLK      479
pbp2b_32494      YFAGSAPITSWYG-AYGSFNIDATEALEYSSNIYMVKIALGLLGQTYSANMYLNDGDTLK      479
pbp2b_22715      YFAGSAPITSWYG-AYDSFNIDATEALEYSSNIYMVKIALGLLGQTYSANMYLNDGDTLK      479
                 ***********:  **.*************** **************************.

pbp2b_32098      NAMTKLRSTFAEYGLGASTGIDLPLESTGFLPEEYSTANFIMNAFGQFDNYTPMQMAQYV      538
pbp2b_33329      NAMTKLRSTFAEYGLGASTGIDLPLESTGFLPEEYSTANFIMNAFGQFDNYTPMQMAQYV      538
pbp2b_37647      NAMTKLRSTFAEYGLGASTGIDLPLESIGFLPEEYSTANFITNAFGQFDNYTPMQMAQYA      538
pbp2b_40085      NAMTKLRSTFAEYGLGASTGIDLPLESIGFLPEEYSTANFITNAFGQFDNYTPMQMAQYA      538
pbp2b_40484      NAMTKLRSTFAEYGLGASTGIDLPLESIGFLPEEYSTANFITNAFGQFDNYTPMQMAQYA      538
pbp2b_40747      NAMTKLRSTFAEYGLGASTGIDLPLESIGFLPEEYSTANFITNAFGQFDNYTPMQMAQYA      538
pbp2b_41126      NAMTKLRSTFAEYGLGASTGIDLPLESIGFLPEEYSTANFITNAFGQFDNYTPMQMAQYA      538
pbp2b_30190      NAMTKLRSTFAEYGLGASTGIDLPLESTGFLPDKYTTANFITNAFGQFDNYTPMQMAQYA      540
pbp2b_P1/7       NAMTKLRSTFAEYGLGASTGIDLPLESTGFLPDKYTTANFITNAFGQFDNYTPMQMAQYA      539
pbp2b_32494      NAMTKLRSTFAEYGLGASTGIDLPLESTGFLPDKYTTANFITNAFGQFDNYTPMQMAQYA      539
pbp2b_22715      NAMTKLRSTFAEYGLGASTGIDLPLESTGFLPDKYTTANFITNAFGQFDNYTPMQMAQYA      539
                 *************************** ****::*:***** *****************.

pbp2b_32098      ATVANNGKRISPHLVEGIYGNNPQGGLGDLIETVSGKEMNQVNISADEMALLRQGFYQLV      598
pbp2b_33329      ATVANNGKRISPHLVEGIYGNNPQGGLGDLIETVSGKEMNQVNISADEMALLRQGFYQLV      598
pbp2b_37647      ATIANNGTRISPHLVEGIYGNNAQGGLGELIETVSGKEMNQVNISAEEMSLLRQGFYQVV      598
pbp2b_40085      ATIANNGTRISPHLVEGIYGNNAQGGLGELIETVSGKEMNQVNISAEEMSLLRQGFYQVV      598
pbp2b_40484      ATIANNGTRISPHLVEGIYGNNAQGGLGELIETVSGKEMNQVNISAEEMSLLRQGFYQVV      598
pbp2b_40747      ATIANNGTRISPHLVEGIYGNNAQGGLGELIETVSGKEMNQVNISAEEMSLLRQGFYQVV      598
pbp2b_41126      ATIANNGTRISPHLVEGIYGNNAQGGLGELIETVSGKEMNQVNISAEEMSLLRQGFYQVV      598
pbp2b_30190      ATIANNGTRISPHLVEGIYGNNAQGGLGELIETVSGKEMNQVNISAEDMSLLRQGFYQVV      600
pbp2b_P1/7       ATIANNGTRISPHLVEGIYGNNAQGGLGELIETVSGKEMNQVNISAEDMSLLRQGFYQVV      599
pbp2b_32494      ATIANNGTRISPHLVEGIYGNNAQGGLGELIETVSGKEMNQVNISAEDMSLLRQGFYQVV      599
pbp2b_22715      ATIANNGTRISPHLVEGIYGNNAQGGLGELIETVSGKEMNQVNISAEDMSLLRQGFYQVV      599
                 **:****.************** *****:*****************::*:********:*
```

```
pbp2b_32098      NGGGRFNTGSAIGRGAAVTISAKTGTAETYTTTPSGEVVTAVNTNVVAYAPSDNPQIAVA      658
pbp2b_33329      NGGGRFNTGSAIGRGAAVTISAKTGTAETYTTTPSGEVVTAVNTNVVAYAPSDNPQIAVA      658
pbp2b_37647      NGSGRFNTGSAIGRGAAVTISAKTGRAETYTTTASGDVVTAVNTNVVAYAPSDNPQIAVA      658
pbp2b_40085      NGSGRFNTGSAIGRGAAVTISAKTGRAETYTTTASGDVVTAVNTNVVAYAPSDNPQIAVA      658
pbp2b_40484      NGSGRFNTGSAIGRGAAVTISAKTGRAETYTTTASGDVVTAVNTNVVAYAPSDNPQIAVA      658
pbp2b_40747      NGSGRFNTGSAIGRGAAVTISAKTGRAETYTTTASGDVVTAVNTNVVAYAPSDNPQIAVA      658
pbp2b_41126      NGSGRFNTGSAIGRGAAVTISAKTGRAETYTTTASGDVVTAVNTNVVAYAPSDNPQIAVA      658
pbp2b_30190      NGSGRFNTGSAIGRGAAVTISAKTGTAETYTTTASGDMVTAINTNVVAYAPSDNPQIAVA      660
pbp2b_P1/7       NGSGRFNTGSAIGRGAAVTISAKTGTAETYTTTASGDVVTAVNTNVVAYAPSDNPQIAVA      659
pbp2b_32494      NGSGRFNTGSAIGRGAAVTISAKTGTAETYTTTASGDVVTAVNTNVVAYAPSDNPQIAVA      659
pbp2b_22715      NGSGRFNTGSAIGRGAAVTISAKTGTAETYTTTASGDVVTAINTNVVAYAPSDNPQIAVV      659
                 **.********************** ******* **::***:*****************.

pbp2b_32098      VVLPNLTNENST-TKAITTEIINLYNSLYPMN  689
pbp2b_33329      VVLPNLTNENST-TKAITTEIINLYNSLYPMN  689
pbp2b_37647      VVLPNLTNQSSTTTNTITQEIINLYQSLHPMN  690
pbp2b_40085      VVLPNLTNQSSTTTNTITQEIINLYQSLHPMN  690
pbp2b_40484      VVLPNLTNQSSTTTNTITQEIINLYQSLHPMN  690
pbp2b_40747      VVLPNLTNQSSTTTNTITQEIINLYQSLHPMN  690
pbp2b_41126      VVLPNLTNQSSTTTNTITQEIINLYQSLHPMN  690
pbp2b_30190      VVLPNLTNQSSTTTKTITQEIINLYQSLHPMN  692
pbp2b_P1/7       VVLPNLTNQSSTTTKTITQEIINLYQSLHPMN  691
pbp2b_32494      VVLPNLTNQSSTTTKTITQEIINLYQSLHPMN  691
pbp2b_22715      VVLPNLTNQSSTTTKTITQEIINLYQSLHPMN  691
                 ********:.** *::** ******:**:***
```
