# Supplementary material for: Non-Penicillin-Susceptible Streptococcus suis Isolated from Humans
Source: Pathogens. 2021 Sep 13;10(9):1178. doi: 10.3390/pathogens10091178 (PMC8471365; doi:10.3390/pathogens10091178)
Supplement: Supplementary file 1 [file pathogens-10-01178-s001.zip › Supplementary file 5_pbp2X alignment result.pdf]

Alignment of pbp2X sequence

```
pbp2x_32098      MPRRNNKLLRYVLKKRYVPSKNRRRVGQSLLLLTVFVFFLFLINFAYIIGTDSKFGVDLS      60
pbp2x_33329      MPRRNNKLLRYVLKKRYVPSKNRRRVGQSLLLLTVFVFFLFLINFAYIIGTDSKFGVDLS      60
pbp2x_30190      MPRRNNKLLRYVLKKRYVPSKNRRRVGQSLLLLTVFVFFLFLINFAYIIGTDSKFGVDLS      60
pbp2x_32494      MPRRNNKLLRYVLKKRYVPSKNRRRVGQSLLLLTVFVFFLFLINFAYIIGTDSKFGVDLS      60
pbp2x_P1-7       MPRRNNKLLRYVLKKRYVPSKNRRRVGQSLLLLTVFVFFLFLINFAYIIGTDSKFGVDLS      60
pbp2x_37647      MPRRNNKLLRYVLKKRYIPSKNRRRVGQSLLLLTVFVFFLFLINFAYIIGTDSKFGVDLS      60
pbp2x_40085      MPRRNNKLLRYVLKKRYIPSKNRRRVGQSLLLLTVFVFFLFLINFAYIIGTDSKFGVDLS      60
pbp2x_41126      MPRRNNKLLRYVLKKRYIPSKNRRRVGQSLLLLTVFVFFLFLINFAYIIGTDSKFGVDLS      60
pbp2x_22715      MPRRNNKLLRYVLKKRYVPSKNRRRVGQSLLLLTVFVFFLFLINFAYIIGTDSKFGVDLS      60
pbp2x_40484      MPRRNNKLLRYVLKKRYVPSKNRRRVGQSLLLLTVFVFFLFLINFAYIIGTDSKFGVDLS      60
pbp2x_40747      MPRRNNKLLRYVLKKRYVPSKNRRRVGQSLLLLTVFVFFLFLINFAYIIGTDSKFGVDLS      60
                 *****************:******************************************

pbp2x_32098      ERADAVHQREVTVQAKRGTIYDRTGIPIAEDSTTYTVYAIIDTEYVSELKKVLYVQSSQF      120
pbp2x_33329      ERADAVHQREVTVQAKRGTIYDRTGIPIAEDSTTYTVYAIIDTEYVSELKKVLYVQSSQF      120
pbp2x_30190      ERADAVHQREVTVQAKRGTIYDRTGIPIAEDSTTYTVYAIIDTEYVSELKKVLYVQSSQF      120
pbp2x_32494      ERADAVHQREVIVQAKRGTIYDRTGIPIAEDSTTYTVYAIIDTEYVSELKKVLYVQSSQF      120
pbp2x_P1-7       ERADAVHQREVIVQAKRGTIYDRTGIPIAEDSTTYTVYAIIDTEYVSELKKVLYVQSSQF      120
pbp2x_37647      ERADAVHQREVTVQAKRGTIYDRTGIPIAEDSTTYTVYAIIDTEYVSELKKVLYVQSSQF      120
pbp2x_40085      ERADAVHQREVTVQAKRGTIYDRTGIPIAEDSTTYTVYAIIDTEYVSELKKVLYVQSSQF      120
pbp2x_41126      ERADAVHQREVTVQAKRGTIYDRTGIPIAEDSTTYTVYAIIDTEYVSELKKVLYVQSSQF      120
pbp2x_22715      ERADAVHQREVTVQAKRGTIYDRTGIPIAEDSTTYTVYAIIDTEYVSEFQEILYVESSQF      120
pbp2x_40484      ERADAVHQREVTVQAKRGTIYDRTGIPIAEDSTTYTVYAIIDTEYVSEFQEILYVESSQF      120
pbp2x_40747      ERADAVHQREVTVQAKRGTIYDRTGIPIAEDSTTYTVYAIIDTEYVSEFQEILYVESSQF      120
                 *********** ************************************::::***:****

pbp2x_32098      SKVAEIFKKHLGMETAYVMEQLNQPELKQVYFGTLGKNISYSTMSSIQEEMKAAGIEGIA      180
pbp2x_33329      SKVAEIFKKHLGMETAYVMEQLNQPELKQVYFGTLGKNISYSTMSSIQEEMKAAGIEGIA      180
pbp2x_30190      SKVAEIFKKHLGMETAYVMEQLNQPELKQVYFGTLGKNISYSTMSSIQEEMKAAGIEGIA      180
pbp2x_32494      SKVAEIFKKHLGMETDYVMEQLNQPELKQVYFGTLGKNISYSTMSSIQEEMKAAGIEGIA      180
pbp2x_P1-7       SKVAEIFKKHLGMETDYVMEQLNQPELKQVYFGTLGKNISYSTMSSIQEEMKAAGIEGIA      180
pbp2x_37647      SKVAEIFKKHLGMETDYVMEQLNQPKLKQVYFGTLGKNISYSTMSSIQEEMKAAGIEGIA      180
pbp2x_40085      SKVAEIFKKHLGMETDYVMEQLNQPKLKQVYFGTLGKNISYSTMSSIQEEMKAAGIEGIA      180
pbp2x_41126      SKVAEIFKKHLGMETDYVMEQLNQPKLKQVYFGTLGKNISYSTMSSIQEEMKAAGIEGIA      180
pbp2x_22715      HKVADILNKYLGMETDYVNKQLNQPDLKQVYFGTLGKNISYSTMTSIQEEMKAAGIKGIA      180
pbp2x_40484      HKVADILNKYLGMETDYVNKQLNQPDLKQVYFGTLGKNISYSTMTSIQEEMKAAGIKGIA      180
pbp2x_40747      HKVADILNKYLGMETDYVNKQLNQPDLKQVYFGTLGKNISYSTMTSIQEEMKAAGIKGIA      180
                  ***:*::*:***** ** :*****.******************:***********:***

pbp2x_32098      FNASPGRMYPNGNFSSIFVGLANPVENKDGSHSLQGVSGLEYYLNDILAGQDGKVIYEKD      240
pbp2x_33329      FNASPGRMYPNGNFSSIFVGLANPVENKDGSHSLQGVSGLEYYLNDILAGQDGKVIYEKD      240
pbp2x_30190      FNASPGRMYPNGNFASIFVGLANPVENKDGSHSLQGVSGLEYYLNDILAGQDGKVIYEKD      240
pbp2x_32494      FNASPGRMYPNGNFASIFVGLANLVENKDGSHSLQGVSGLEYYLNDILAGQDGKVIYEKD      240
pbp2x_P1-7       FNASPGRMYPNGNFASIFVGLANLVENKDGSHSLQGVSGLEYYLNDILAGQDGKVIYEKD      240
pbp2x_37647      FDASPGRMYSNGNFASIFVGLANPVENKDGSYSLQGVSGLEYYLNDILAGQDGKVIYEKD      240
pbp2x_40085      FDASPGRMYSNGNFASIFVGLANPVENKDGSYSLQGVSGLEYYLNDILAGQDGKVIYEKD      240
pbp2x_41126      FDASPGRMYSNGNFASIFVGLANPVENKDGSYSLQGVSGLEYYLNDILAGQDGKVIYEKD      240
pbp2x_22715      FNASPGRMYPNGNFASIFVGLANLIENKDGSHSLQGMSGMEYYLNDILAGQDGKVIYEKD      240
pbp2x_40484      FNASPGRMYPNGNFASIFVGLANLIENKDGSHSLQGMSGMEYYLNDILAGQDGKVIYEKD      240
pbp2x_40747      FNASPGRMYPNGNFASIFVGLANLIENKDGSHSLQGMSGMEYYLNDILAGQDGKVIYEKD      240
                 *:******* ****:******** :******:****:**:********************
```

```
pbp2x_32098      SQGRVLPGTEKVETETVNGQDVYTTISADLQRSLETNMNTFFDNAKGRYANATLVSAKTG      300
pbp2x_33329      SQGRVLPGTEKVETETVNGQDVYTTISADLQRSLETNMNTFFDNAKGRYANATLVSAKTG      300
pbp2x_30190      SQGRVLPGTEKVETETVNGQDVYTTISADLQRSLETNMNTFFDNAKGRYANATLVSAKTG      300
pbp2x_32494      SQGRVLPGTEKVETETVNGQDVYTTISADLQRSLETNMNTFFDNAKGRYANATLVSAKTG      300
pbp2x_P1-7       SQGRVLPGTEKVETETVNGQDVYTTISADLQRSLETNMNTFFDNAKGRYANATLVSAKTG      300
pbp2x_37647      SQGRVLPGTEKVETETVNGQDVYTTISADLQRSLETNMNTFFDNAKGRYANATLVSAKTG      300
pbp2x_40085      SQGRVLPGTEKVETETVNGQDVYTTISADLQRSLETNMNTFFDNAKGRYANATLVSAKTG      300
pbp2x_41126      SQGRVLPGTEKVETETVNGQDVYTTISADLQRSLETNMNTFFDNAKGRYANATLVSAKTG      300
pbp2x_22715      SQGRILPGTESVEQETVNGQDVYTTISADLQRSLEINMNTFFDTAKGKYANATLVSAKTG      300
pbp2x_40484      SQGRILPGTESVEQETVNGQDVYTTISADLQRSLETNMNTFFDTAKGKYANATLVSAKTG      300
pbp2x_40747      SQGRILPGTESVEQETVNGQDVYTTISADLQRSLETNMNTFFDTAKGKYANATLVSAKTG      300
                 ****:*****.** ********************** *******.***:***********

pbp2x_32098      EILATTQRPSYDSDTKEGLGQEGLLERSLLYQEAFEPGSTMKVLTVASAIDNGTFDPNAY      360
pbp2x_33329      EILATTQRPSYDSDTKEGLGQEGLLERSLLYQEAFEPGSTMKVLTVASAIDNGTFDPNAY      360
pbp2x_30190      EILATTQRPSYDSDTKEGLGQEGLLERSLLYQEAFEPGSTMKVLTVASAIDNGTFDPNAY      360
pbp2x_32494      EILATTQRPSYDSDTKEGLGQEGLLERSLLYQEAFEPGSTMKVLTVASAIDNGTFDPNAY      360
pbp2x_P1-7       EILATTQRPSYDSDTKEGLGQEGLLERSLLYQEAFEPGSTMKVLTVASAIDNGTFDPNAY      360
pbp2x_37647      EILATTQRPSYDSDTKEGLGQEGLLERSLLYQEAFEPGSTMKVLTVASAIDNGTFDPNAY      360
pbp2x_40085      EILATTQRPSYDSDTKEGLGQEGLLERSLLYQEAFEPGSTMKVLTVASAIDNGTFDPNAY      360
pbp2x_41126      EILATTQRPSYDSDTKEGLGQEGLLERSLLYQEAFEPGSTMKVLTVASAIDNGTFDPNAY      360
pbp2x_22715      EILATTQRPSYDSDTKEGLGEEGLLERSLLYQEAFEPGSTMKVLTVASAIDNGTFDPNAY      360
pbp2x_40484      EILATTQRPSYDSDTKEGLGEEGLLERSLLYQEAFEPGSTMKVLTVASAIDNGTFDPNAY      360
pbp2x_40747      EILATTQRPSYDSDTKEGLGEEGLLERSLLYQEAFEPGSTMKVLTVASAIDNGTFDPNAY      360
                 ********************:***************************************

pbp2x_32098      YTNNEYVIADAKINDWTLNAGYSAVTLNYAQGFSLSSNVGMTLLQQQMGDEKWLNYLAKF      420
pbp2x_33329      YTNNEYVIADAKINDWTLNAGYSAVTLNYAQGFSLSSNVGMTLLQQQMGDEKWLNYLAKF      420
pbp2x_30190      YTNNEYVIADAKINDWTLNAGYSAVTLNYAQGFSLSSNVGMTLLQQQMGDEKWLNYLTKF      420
pbp2x_32494      YTNNEYVIADAKINDWTLNAGYSAVTLNYAQGFSLSSNVGMTLLQQQMGDEKWLNYLTKF      420
pbp2x_P1-7       YTNNEYVIADAKINDWTLNAGYSAVTLNYAQGFSLSSNVGMTLLQQQMGDEKWLNYLTKF      420
pbp2x_37647      YTNNEYVIADAKINDWTLNAGYSAVTLNYAQGFSLSSNVGMTLLQQQMGDEKWLNYLTKF      420
pbp2x_40085      YTNNEYVIADAKINDWTLNAGYSAVTLNYAQGFSLSSNVGMTLLQQQMGDEKWLNYLTKF      420
pbp2x_41126      YTNNEYVIADAKINDWTLNAGYSAVTLNYAQGFSLSSNVGMTLLQQQMGDEKWLNYLTKF      420
pbp2x_22715      YTNNEYVIADAKINDWTLNAGQSAVTLNFAQGFSLSSNVGMTLLQQEMGDEKWLNYLAKF      420
pbp2x_40484      YTNNEYVIADAKINDWTLNAGQSAVTLNFAQGFSLSSNVGMTLLQQEMGDEKWLNYLAKF      420
pbp2x_40747      YTNNEYVIADAKINDWTLNAGQSAVTLNFAQGFSLSSNVGMTLLQQEMGDEKWLNYLAKF      420
                 ********************* ******:*****************:**********:**

pbp2x_32098      RFGYPTRFGMGDEAPGMVPEDNIVSIAMSSFGQGISANQAQMLRSFTSIANNGIMLEPKF      480
pbp2x_33329      RFGYPTRFGMGDEAPGMVPEDNIVSIAMSSFGQGISANQAQMLRSFTSIANNGIMLEPKF      480
pbp2x_30190      RFGYPTRFGMGDEAPGMVPEDNIVSIAMSSFGQGISANQAQMLRSFTSIANNGIMLEPKF      480
pbp2x_32494      RFGYPTRFGMGDEAPGMVPEDNIVSIAMSSFGQGISANQAQMLRSFTAIANNGIMLEPKF      480
pbp2x_P1-7       RFGYPTRFGMGDEAPGMVPEDNIVSIAMSSFGQGISANQAQMLRSFTAIANNGIMLEPKF      480
pbp2x_37647      RFGYPTRFGMGDEAPGMVPEDNIVSIAMSTFGQGISANQAQMLRSFTAIANNGIMLEPKF      480
pbp2x_40085      RFGYPTRFGMGDEAPGMVPEDNIVSIAMSTFGQGISANQAQMLRSFTAIANNGIMLEPKF      480
pbp2x_41126      RFGYPTRFGMGDEAPGMVPEDNIVSIAMSTFGQGISANQAQMLRSFTAIANNGIMLEPKF      480
pbp2x_22715      RYGYPTRFGMGDEAPGMVPEDNIVSIAMSTFGQGISANQAQMLRSFTAIANNGVMLEPKF      480
pbp2x_40484      RYGYPTRFGMGDEAPGMVPEDNIVSIAMSTFGQGISANQAQMLRSFTAIANNGVMLEPKF      480
pbp2x_40747      RYGYPTRFGMGDEAPGMVPEDNIVSIAMSTFGQGISANQAQMLRSFTAIANNGVMLEPKF      480
                 *:***************************:*****************:******:*****

pbp2x_32098      ISALYDPNTDTARVSSKEEVGNPVSEQAADDTLRYMINVGTDPVYGTLYSHDLQGPAIQV      540
pbp2x_33329      ISALYDPNTDTARVSSKEEVGNPVSEQAADDTLRYMINVGTDPVYGTLYSHDLQGPAIQV      540
pbp2x_30190      ISALYDPNTDTARVSSKEEVGNPVSEQAADDTLRYMINVGTDPVYGTLYSHDLQGPAIQV      540
pbp2x_32494      ISALYDPNTDTARVSSKEEVGNPVSEQAADDTLRYMINVGTDPVYGTLYSHDLQGPAIQV      540
pbp2x_P1-7       ISALYDPNTDTARVSSKEEVGNPVSEQAADDTLRYMINVGTDPVYGTLYSHDLQGPAIQV      540
pbp2x_37647      ISALYNPNTDTARVSSKEEVGNPVSEQAADDTLRYMINVGTDPVYGTLYSHDLQGPAIQV      540
pbp2x_40085      ISALYNPNTDTARVSSKEEVGNPVSEQAADDTLRYMINVGTDPVYGTLYSHDLQGPAIQV      540
pbp2x_41126      ISALYNPNTDTARVSSKEEVGNPVSEQAADDTLRYMINVGTDPVYGTLYSHDLQGPAIQV      540
pbp2x_22715      ISALYDPNTDSARIAAKEEVGNPVSEQAADDTLRYMINVGTDPVFGTLYSHDVQGPAIQV      540
pbp2x_40484      ISALYDPNTDSARIAAKEEVGNPVSEQAADDTLRYMINVGTDPVFGTLYSHDVQGPAIQV      540
pbp2x_40747      ISALYDPNTDSARIAAKEEVGNPVSEQAADDTLRYMINVGTDPVFGTLYSHDVQGPAIQV      540
                 *****:****:**:::****************************:********:*******
```

```
pbp2x_32098      DGYDIAVKSGSAEIASEDGQGYIKGAYTNSVVAMIPAENPEFIMYVTIRQPEVLSVLSWD      600
pbp2x_33329      DGYDIAVKSGSAEIASEDGQGYIKGAYTNSVVAMIPAENPEFIMYVTIRQPEVLSVLSWD      600
pbp2x_30190      DGYDIAVKSGTAEIASEDGQGYIKGAYINSVVAMIPAEDPEFIMYVTIRQPEVLNVLSWR      600
pbp2x_32494      DGYDIAVKSGTAEIASEDGQGYIKGAYINSVVAMIPAEDPEFIMYVTIRQPEVLNVLSWR      600
pbp2x_P1-7       DGYDIAVKSGTAEIASEDGQGYIKGAYINSVVAMIPAEDPEFIMYVTIRQPEVLNVLSWR      600
pbp2x_37647      DGYDIAVKSGSAEIASEDGQGYIKGAYTNSVVAMIPAEDPEFIMYVTIRQPEVLNVLSWR      600
pbp2x_40085      DGYDIAVKSGSAEIASEDGQGYIKGAYTNSVVAMIPAEDPEFIMYVTIRQPEVLNVLSWR      600
pbp2x_41126      DGYDIAVKSGSAEIASEDGQGYIKGAYTNSVVAMIPAEDPEFIMYVTIRQPEVLNVLSWR      600
pbp2x_22715      EGYDIAMKSGSAEIAGEDGLGYIKDAYTNSVVAMIPAEDPEFIMYVTIRQPEVLSVLSWN      600
pbp2x_40484      EGYDIAMKSGSAEIAGEDGLGYIKDAYTNSVVAMIPAEDPEFIMYVTIRQPEVLNVLSWR      600
pbp2x_40747      EGYDIAMKSGSAEIAGEDGLGYIKDAYTNSVVAMIPAEDPEFIMYVTIRQPEVLNVLSWR      600
                 :*****:***:****.*** ****.** **********:***************.****

pbp2x_32098      SIISPTLKEAMLLKDNLNLDAPAPSLTYVEKETDYKLPDLIGKAPGESAEELRRQLVQPV      660
pbp2x_33329      SIISPTLKEAMLLKDNLNLDAPAPSLTYVEKETDYKLPDLIGKAPGESAEELRRQLVQPV      660
pbp2x_30190      DIVSPTLKEAMLLKDNLNLDSPAPALARVANETEYQLPDLIGKAPGESAEELRRQLVQPV      660
pbp2x_32494      DIVSPTLKEAMLLKDNLNLDSPAPALARVANETEYQLPDLIGKAPGESAEELRRQLVQPV      660
pbp2x_P1-7       DIVSPTLKEAMLLKDNLNLDSPAPALARVANETEYQLPDLIGKAPGESAEELRRQLVQPV      660
pbp2x_37647      DIVSPTLKEAMLLKDNLNLDSPAPALAQVANETEYQLPDLIGKAPGESAEELRRQLVQPV      660
pbp2x_40085      DIVSPTLKEAMLLKDNLNLDSPAPALAQVANETEYQLPDLIGKAPGESAEELRRQLVQPV      660
pbp2x_41126      DIVSPTLKEAMLLKDNLNLDSPAPALAQVANETEYQLPDLIGKAPGESAEELRRQLVQPV      660
pbp2x_22715      TIVSPTLKEAMLLKDNLNLDVPASNLSLLTNETEYKLPDIVGKAPGEIAEELRRNLVQPV      660
pbp2x_40484      DIVSPTLKEAMLLKDNLNLDSPAPALAQVANETEYQLPDLIGKAPGESAEELRRQLVQPV      660
pbp2x_40747      DIVSPTLKEAMLLKDNLNLDSPAPALAQVANETEYQLPDLIGKAPGESAEELRRQLVQPV      660
                  *:***************** **  *: : :**:*:***::****** ******:*****

pbp2x_32098      ILGNGAEIKKVSVEKGSNVIANQQVLLLTNKFEEMPDLYAITKENMDIFAEWTGIEVTYK      720
pbp2x_33329      ILGNGAEIKKVSVEKGSNVIANQQVLLLTNKFEEMPDLYAITKENMDIFAEWTGIEVTYK      720
pbp2x_30190      ILGNGAEIKKVSVEKGSKVIANQQVLLLTNKFEEMPDLYAITKENMDIFAEWTGIEVTYK      720
pbp2x_32494      ILGNGAEIKKVSVEKGSKVIANQQVLLLTNKFEEMPDLYAITKENMDIFAEWTGIEVTYK      720
pbp2x_P1-7       ILGNGAEIKKVSVEKGSKVIANQQVLLLTNKFEEMPDLYAITKENMDIFAEWTGIEVTYK      720
pbp2x_37647      ILGNGAEIKKVSVEKGSNVIANQQVLLLTNKFEEMPDLYAITKENMDIFAEWTGIEVTYK      720
pbp2x_40085      ILGNGAEIKKVSVEKGSNVIANQQVLLLTNKFEEMPDLYAITKENMDIFAEWTGIEVTYK      720
pbp2x_41126      ILGNGAEIKKVSVEKGSNVIANQQVLLLTNKFEEMPDLYAITKENMDIFAEWTGIEVTYK      720
pbp2x_22715      ILGNGSEIKQASVEKGTAVSANQQVLLLTNKFEEMPDLYAVTKENMDLIAEWTGIEVTYE      720
pbp2x_40484      ILGNGAEIKKVSVEKGSNVIANQQVLLLTNKFEEMPDLYAITKENMDIFAEWTGIEVTYK      720
pbp2x_40747      ILGNGAEIKKVSVEKGSNVIANQQVLLLTNKFEEMPDLYAITKENMDIFAEWTGIEVTYK      720
                 *****:***:.*****: * ********************:******::**********:

pbp2x_32098      GTGSKVVDQSVEVGTALNKTKKITITLGD     749
pbp2x_33329      GTGSKVVDQSVEVGTALNKTKKITITLGD     749
pbp2x_30190      GTGSKVVDQSVEVGTALNKTKKITITLGD     749
pbp2x_32494      GTGSKVVDQSVEVGTALNKTKKITITLGD     749
pbp2x_P1-7       GTGSKVVDQSVEVGTALNKTKKITITLGD     749
pbp2x_37647      GTGSKVVDQSVEVGTALNKTEKITITLGD     749
pbp2x_40085      GTGSKVVDQSVEVGTALNKTEKITITLGD     749
pbp2x_41126      GTGSKVVDQSVEVGTALNKTEKITITLGD     749
pbp2x_22715      GQGAKVIDQSVEVGTKLNKTKNITVTLGD     749
pbp2x_40484      GTGSKVVDQSVEVGTALNKTEKITITLGD     749
pbp2x_40747      GTGSKVVDQSVEVGTALNKTEKITITLGD     749
                 * *:**:******** ****::**:****
```
