# Supplementary material for: Non-Penicillin-Susceptible Streptococcus suis Isolated from Humans
Source: Pathogens. 2021 Sep 13;10(9):1178. doi: 10.3390/pathogens10091178 (PMC8471365; doi:10.3390/pathogens10091178)
Supplement: Supplementary file 1 [file pathogens-10-01178-s001.zip › Table S1.pdf]

Table S1. Antimicrobial resistance patterns and the resistance genes of *S. suis* by serotypes and sequence types

Serotype	ST	Macrolide and tetracycline resistance genes profile	Antimicrobial resistance pattern	Phenotype	Isolates
1	105 (n=2)	*ermB+tetO*	ATH+E+T+CD	MDR	2
2	1 (n=143)	*ermB+tetO*	ATH+E+T+CD	MDR	58
			ATH+E+T+CD+C	MDR	32
			T	-	7
			E+T+CD	MDR	4
			ATH+T+CD	MDR	1
			T+CD	-	3
			T+C	-	1
			E+CD	-	1
			T+C+CD	MDR	1
		tetO	T	-	11
			ATH+E+T+CD	MDR	7
			E+T+CD	MDR	3
			ATH+E+T+CD+C	MDR	1
			ATH+T+CD	MDR	1
			T+C+P	MDR	1
			T+CD	-	4
		ermB+tetO+tetW	ATH+E+T+CD	MDR	3
			ATH+E+T+CD+C	MDR	1
			ATH+E+T+CD+P	MDR	1
		ermB+tetM	T	-	1
		unknown	E+CD	-	1
	25 (n=22)	*ermB+tetO*	ATH+E+T+CD+C	MDR	10
			ATH+E+T+CD	MDR	7
			ATH+E+T+CD+C+P	MDR	1
		ermB+tetO+tetW	ATH+E+T+CD+C	MDR	3
		tetL+tetM	ATH+E+T+CD+LEV	MDR	1
	28 (n=9)	*tetO*	ATH+E+T+CD	MDR	1
		ermB+tetO	ATH+E+T+CD	MDR	6
			E+T+CD	MDR	2
	103 (n=7)	*ermB+tetO+tetW*	ATH+E+T+CD	MDR	4
		ermB+tetO	ATH+E+T+CD+C	MDR	2
		ermB	ATH+E+T+CD+C	MDR	1

Serotype	ST	Macrolide and tetracycline resistance genes profile	Antimicrobial resistance pattern	Phenotype	Isolates
2	104 (n=111)	*ermB+tetO*	ATH+E+T+CD	MDR	57
			ATH+E+T+CD+C	MDR	14
			ATH+E+CD	-	1
			ATH+E+T+CD+C+P	MDR	1
			E+T+CD	MDR	4
			T	-	1
			ATH+E+T+CD+P	MDR	1
		tetO	E+T+CD	MDR	11
			ATH+E+T+CD	MDR	4
			ATH+E+T+CD+C+P	MDR	1
		ermB+tetO+tetW	ATH+E+T+CD+C	MDR	8
			ATH+E+T+CD	MDR	4
			ATH+T	-	1
		tetO+tetW	ATH+E+T+CD+C	MDR	1
		ermB	ATH+E+T+CD	MDR	2
	105 (n=5)	*ermB+tetO*	ATH+E+T+CD	MDR	2
			ATH+E+T+C+CD	MDR	2
			ATH+E+T+CD+P	MDR	1
	126 (n=3)	*ermB+tetO*	E+T+CD	MDR	2
		tetO	E+T+CD+C	MDR	1
	144 (n=7)	*tetO*	E+T+CD	MDR	2
		ermB+tetO	ATH+E+T+CD	MDR	4
			ATH+E+T+CD+C	MDR	1
	233 (n=33)	*ermB+tetO*	ATH+E+T+CD+P	MDR	14
			ATH+E+T+CD	MDR	9
			ATH+E+T+CD+C+P	MDR	3
			ATH+E+T+CD+C	MDR	2
			E+T+CD+P	MDR	2
			ATH+E+CD+P	MDR	1
			ATH+E+CD+C+P	MDR	1
		tetO	ATH+E+T+CD	MDR	1

Serotype	ST	Macrolide and tetracycline resistance genes profile	Antimicrobial resistance pattern	Phenotype	Isolates
2	236 (n=1)	*ermB+tetO*	ATH+E+T+CD	MDR	1
	298 (n=1)	unknown	E+CD	-	1
	337 (n=5)	*ermB+tetO*	E+T+CD	MDR	1
		tetO	E+T+CD	MDR	2
			T+CD	-	1
			ATH+E+T	-	1
	379 (n=3)	*ermB+tetO*	ATH+E+T+CD+P	MDR	2
			E+T+CD+P	MDR	1
	380 (n=8)	*ermB+tetO*	ATH+E+T+CD	MDR	6
			ATH+E+T+CD+C	MDR	1
			ATH+E+CD	-	1
	381 (n=4)	*ermB+tetO*	ATH+E+T+CD+C	MDR	3
			ATH+E+T+CD	MDR	1
	382 (n=2)	*ermB+tetO*	ATH+E+T+CD+P	MDR	1
			ATH+E+T+CD+C	MDR	1
	391 (n=9)	*ermB+tetO*	ATH+E+T+CD	MDR	6
			ATH+E+T+CD+C	MDR	1
			E+T+CD	MDR	1
		ermB	ATH+E+T+CD	MDR	1
	392 (n=4)	*ermB+tetO*	ATH+E+T+CD	MDR	2
			ATH+E+T+CD+C	MDR	1
			E+T+CD	MDR	1
	393 (n=1)	*ermB+tetO*	ATH+E+T+CD+C+P	MDR	1
	395 (n=1)	*ermB+tetO*	ATH+E+T+CD+C	MDR	1
	512 (n=8)	*ermB+tetO*	ATH+E+T+CD	MDR	5
			E+T+CD	MDR	2
			E+T+CD+C	MDR	1

Serotype	ST	Macrolide and tetracycline resistance genes profile	Antimicrobial resistance pattern	Phenotype	Isolates
2	513 (n=1)	*ermB+tetO*	ATH+E+T+CD	MDR	1
	514 (n=2)	*ermB+tetO*	ATH+E+T+CD	MDR	1
			ATH+E+T+CD+C	MDR	1
	515 (n=1)	*ermB*	ATH+E+T+CD	MDR	1
	516 (n=1)	*ermB+tetO*	ATH+E+T+CD+C+LEV	MDR	1
4	94 (n=1)	*ermB+tetO*	ATH+E+T+CD+C	MDR	1
5	235 (n=1)	*ermB+tetO*	ATH+E+T+CD+C+P	MDR	1
	221 (n=1)	*ermB+tetO*	ATH+E+T+CD+C	MDR	1
9	16 (n=1)	tetW	ATH+E+T+CD	MDR	1
14	11 (n=2)	*tetO*	E+T+CD	MDR	1
		unknown	T+CD	-	1
	105 (n=44)	*ermB+tetO*	ATH+E+T+CD	MDR	27
			ATH+E+T+C+CD	MDR	11
			ATH+E+T+C+CD+P	MDR	1
			ATH+E+CD	-	1
		ermB	ATH+E+T+CD	MDR	1
		tetO	ATH+E+T+CD	MDR	2
		tetL+tetM	ATH+E+T+CD	MDR	1
24	237 (n=1)	*ermB+tetO*	ATH+E+T+CD+C	MDR	1
	221 (n=2)	*ermB+tetO*	ATH+E+T+CD	MDR	1
			ATH+E+T+CD+C+P	MDR	1
	234 (n=1)	*ermB+tetO*	ATH+E+T+CD+P	MDR	1
**Total**					**448**

Abbreviations; Cefepime; CPM, ceftriaxone; CRO, levofloxacin; LEV, chloramphenicol; C, tetracycline; T, azithromycin; ATH, clindamycin; C, erythromycin; E and penicillin; P
