# Supplementary material for: Non-Penicillin-Susceptible Streptococcus suis Isolated from Humans
Source: Pathogens. 2021 Sep 13;10(9):1178. doi: 10.3390/pathogens10091178 (PMC8471365; doi:10.3390/pathogens10091178)
Supplement: Supplementary file 1 [file pathogens-10-01178-s001.zip › Table S3.pdf]

Table S3 PCR primers for detection of tetracycline and macrolide resistance genes.

Primer	Targeted gene	Sequences (5'-3')	Annealing temperature (°C)	Amplicon size (bp)
*tet*A-FW	*tet*A	GCGCGATCTGGTTCACTCG	61	164
*tet*A-RV		AGTCGACAGYRGCGCCGGC		
*tet*B-FW	*tet*B	TACGTGAATTTATTGCTTCGG	59	206
*tet*B-RV		ATACAGCATCCAAAGCGCAC		
*tet*C-FW	*tet*C	GCGGGATATCGTCCATTCCG	68	207
*tet*C-RV		GCGTAGAGGATCCACAGGACG		
*tet*D-FW	*tet*D	GGAATATCTCCCGGAAGCGG	68	187
*tet*D-RV		CACATTGGACAGTGCCAGCAG		
*tet*E-FW	*tet*E	GTTATTACGGGAGTTTGTTGG	61	199
*tet*E-RV		AATACAACACCCACACTACGC		
*tet*G-FW	*tet*G	GCAGAGCAGGTCGCTGG	65	134
*tet*G-RV		CCYGCAAGAGAAGCCAGAAG		
*tet*H-FW	*tet*H	CAGTGAAAATTCACTGGCAAC	61	185
*tet*H-RV		ATCCAAAGTGTGGTTGAGAAT		
*tet*J-FW	*tet*J	CGAAAACAGACTCGCCAATC	61	184
*tet*J-RV		TCCATAATGAGGTGGGGC		
*tet*K-FW	*tet*K	TCGATAGGAACAGCAGTA	55	169
*tet*K-RV		CAGCAGATCCTACTCCTT		
*tet*L-FW	*tet*L	TCGTTAGCGTGCTGTCATTC	55	267
*tet*L-RV		GTATCCCACCAATGTAGCCG		
*tet*V-FW	*tetV*	GCCTACGGTTTCATCCTGGC	65	351
*tet*V-RV		CGAGACCACCTTCGACAGCG		
*tet*Y-FW	*tet*Y	ATTTGTACCGGCAGAGCAAAC	68	181
*tet*Y-RV		GGCGCTGCCGCCATTATGC		
*tet*Z-FW	*tet*Z	CCTTCTCGACCAGGTCGG	61	204
*tet*Z-RV		ACCCACAGCGTGTCCGTC		
*tet*A(P)-FW	*tet*A(P)	CTTGGATTGCGGAAGAAGAG	55	676
*tet*A(P)-RV		ATATGCCCATTTAACCACGC		
*tet*30-FW	*tet*30	CATCTTGGTCGAGGTGACTGG	68	210
*tet*30-RV		ACGAGCACCCAGCCGAGC		
*tet*31-FW	*tet*31	CAATCACGCCCAAAAGAA	53	564
*tet*31-RV		TGTGCCATCCCAGTTTGT		
*tet*33-FW	*tet*33	ATGCGGTTCCGCTGAA	54	784
*tet*33-RV		GGAAAATGCGTCAGTGACAA		
*tet*35-FW	*tet*35	ATGCGCAAGACCGTCCTAC	54	
*tet*35-RV		CACACACTAGTAACGGTCGAA		
*tet*38-FW	*tet*38	ATGAATGTTGAATATTCTAA	42	106
*tet*38-RV		TGGCTACAGAAATCAAT		
*tet*39-FW	*tet*39	CTCCTTCTCTATTGTGGCTA	47	701
*tet*39-RV		CACTAATACCTCTGGACATCA		
*tet*40-FW	*tet*40	CGGAGGAAGAGGACAAACCC	56	446
*tet*40-RV		TAAGCCGCTGCCGATAAGAC		
*tet*41-FW	*tet*41	AATGCGATCAATTTCCGCCG	55	166
*tet*41-RV		CGGCGAACAGCAGATTAACG		
*tet*42-FW	*tet*42	TCTCGAGGATCACGAACCCT	55	128
*tet*42-RV		ACTGGGACTCGATACACCCA		
*tet*45-FW	*tet*45	GCTGAGCCATCCACTCATTT	63	107
*tet*45-RV		TTTCCTCTTGAGCGTTTATGC		
*tet*AB(46)-FW	*tet*AB(46)	GCTTCTTGGACCTTGACGGA	55	580

*tet*AB(46)-RV		GTTCCTGACTCATGGCCACA		
*tet*47-FW	*tet*47	GCGTTTGGCGTGGGTTTAAT	55	627
*tet*47-RV		GACCCCTGTGGCATTGGTTA		
*tcr*3-FW	*tcr*3	CGCTCAGTTCGACAAGACCT	54	399
*tcr*3-RV		GTCTCCATCGAGTTCGCCAT		
*otr*B-FW	*otr*B	CCGACATCTACGGGCGCAAGC	55	947
*otr*B-RV		GGTGATGACGGTCTGGGACAG		
*otr*C-FW	*otr*C	ATGAAGTTCCGCCGAATGNA	55	1860
*otr*C-RV		TCAGGTCTTCTTGCGGAACTT		
*tet*Q-FW	*tet*Q	AGAATCTGCTGTTTGCCAGTG	56	169
*tet*Q-RV		CGGAGTGTCAATGATATTGCA		
*tet*T-FW	*tet*T	AAGGTTTATTATATAAAAGTG	46	169
*tet*T-RV		AGGTGTATCTATGATATTTAC		
*tet*B(P)-FW	*tet*B(P)	AAAACTTATTATATTATAGTG	46	169
*tet*B(P)-RV		TGGAGTATCAATAATATTCAC		
*tet*32-FW	*tet*32	GAACCAGATGCTGCTCTT	57	620
*tet*32-RV		CATAGCCACGCCCACATGAT		
*tet*36-FW	*tet*36	TTTCTGGCAGAGGTAGAACG	57	250
*tet*36-RV		TTAATTCCTTGCCTTCAACG		
*tet*44-FW	*tet*44	AAAATAATCAACATTGGTATTCTTGCTCA	56	1927
*tet*44-RV		TAGTAACTTAATTTTCTTTTTTATTAAACA TATGGCG		
*otr*A-FW	*otr*A	GAACACGTACTGACCGAGAAG	55	778
*otr*A-RV		CAGAAGTAGTTGTGCGTCCG		
*tet*X-FW	*tet*X	GAAAGAGACAACGACCGAGAG	56.5	131
*tet*X-RV		ACACCCATTGGTAAGGCTAAG		
*tet*34-FW	*tet*34	ATACGGGGATGCAAACTTCA	53	729
*tet*34-RV		ACGAGTGAGCTCTGATGTCTCTT		
*tet*37-FW	*tet*37	ATGGTTCGCTATTACTCTAAC	45	177
*tet*37-RV		ATCAGTCTCATATTTCGACA		
*tet*U-FW	*tet*U	ATGCAGCTAAGACGTGGC	54	317
*tet*U-RV		TTATTCGGTATCACTTCTCTGTC		
erm(B)-F	*erm*(B)	TGGTATTCCAAATGCGTAATG	62	745
erm(B)-R		CTGTGGTATGGCGGGTAAGT		
mef(A/E)-F	*mef*(A/E)	CAATATGGGCAGGGCAAG	62	317
mef(A/E)-R		AAGCTGTTCCAATGCTACGG		
msr(D)-F	*msr*(D)	TTGGACGAAGTAACTCTG	50	370
msr(D)-R		GCTTGGCTCTTACGTTC		
